# METTL3/ALKBH5‐Mediated N6‐Methyladenosine Modification Drives Macrophage M1 Polarization via the SLC15A3‐TASL‐IRF5 Signaling Axis in Psoriasis

**DOI:** 10.1002/advs.202501408

**Published:** 2025-07-18

**Authors:** Tao Huang, Shijun Chen, Ke Ding, Liyan Yuan, Weiqi Lv, Kechen Chen, Yuchen Liu, Dongzhao Ma, Xin Zhang, Xiaobo Wang, Guanzheng Luo, Bin Yang, Ying Lin, Zhili Rong

**Affiliations:** ^1^ Dermatology Hospital Southern Medical University Guangzhou 510091 China; ^2^ Cancer Research Institute School of Basic Medical Sciences State Key Laboratory of Multi‐organ Injury Prevention and Treatment Guangdong Province Key Laboratory of Immune Regulation and Immunotherapy Southern Medical University Guangzhou 510515 China; ^3^ MOE Key Laboratory of Gene Function and Regulation State Key Laboratory of Biocontrol School of Life Sciences Sun Yat‐sen University Guangzhou Guangdong 510275 China

**Keywords:** macrophage polarization, N6‐methyladenosine, psoriasis, SLC15A3, TASL

## Abstract

Impaired N6‐methyladenosine (m^6^A) modification has been implicated in regulating various inflammatory diseases, but its role in psoriasis remains unclear. Here, m^6^A modification and its methyltransferase METTL3 are revealed to be upregulated in psoriatic macrophages, while the demethylase ALKBH5 is downregulated. Conditional knockout of *Mettl3* in macrophages alleviated psoriasis‐like symptoms in mice, whereas knockout of Alkbh5 exacerbated them. Both in vivo and in vitro, *Mettl3* deficiency inhibited IMQ‐induced M1 macrophage polarization, while *Alkbh5* deficiency promoted M1 polarization. The regulation of macrophage polarization by m^6^A is likely mediated by targeting *Slc15a3*. SLC15A3 enhances the recruitment of TASL, a recently identified endolysosomal IRF5 adaptor, which functions similarly to the IRF3 adaptors STING and MAVS at the endoplasmic reticulum (ER) and mitochondria, respectively, to augment IRF5 signaling via SLC15A4. The findings underscore the critical role of m^6^A RNA modification in psoriasis pathogenesis and unveil a novel regulatory mechanism of TASL‐IRF5 signaling through m^6^A modification, suggesting potential new therapeutic targets for psoriasis treatment.

## Graphical abstract

m^6^A modification stabilizes *Slc15a3* mRNA in macrophages, activating the SLC15A3‐TASL‐IRF5 axis and promoting psoriatic inflammation.

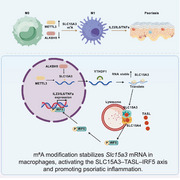

## 1 Introduction

Psoriasis is a complex, chronic inflammatory skin disorder characterized by aberrant immune responses and sustained inflammation.^[^ ^1^ ^]^ Macrophages play a pivotal role in the pathogenesis of psoriasis, as murine studies have demonstrated that their depletion alleviates cutaneous inflammation and normalizes Th1 cytokine levels.^[^ ^2^, ^3^, ^4^ ^]^ This underscores the integral role of macrophages in psoriatic lesion development and maintenance. Patients with psoriasis exhibit elevated circulating monocyte levels,^[^ ^5^, ^6^ ^]^ predominantly skewed toward the pro‐inflammatory M1 phenotype.^[^ ^7^, ^8^ ^]^ Moreover, M1 macrophages and their associated cytokines—including TNFα, IL‐23, and IL‐12—are significantly upregulated in psoriatic lesions, whereas anti‐TNFα therapies have been shown to suppress M1 polarization in affected tissues.^[^ ^9^, ^10^, ^11^ ^]^

Recent studies have identified N⁶‐methyladenosine (m^6^A) RNA modification as a critical regulator of macrophage function.^[^ ^12^, ^13^, ^14^ ^]^ METTL3, a m^6^A writer enzyme, has been shown to enhance M1 polarization by methylating *STAT1* mRNA following IFN‐γ stimulation.^[^ ^12^ ^]^ In the context of LPS/TLR4 signaling, m^6^A modification of *Irakm* mRNA promotes macrophage activation,^[^ ^13^ ^]^ while m^6^A‐mediated repression of *Socs1* expression has been shown to inhibit macrophage activation in other settings.^[^ ^14^ ^]^ Collectively, these findings underscore the context‐dependent roles of m^6^A in macrophage‐mediated immune regulation. However, the functional significance of m^6^A modification in psoriatic macrophages remains largely undefined.

Aberrant activation of endosomal Toll‐like receptors TLR7–9 is recognized as a pathogenic driver in psoriasis. TLR7 agonists have been shown to shift macrophages toward an elevated M1/M2 ratio in psoriatic skin.^[^ ^7^, ^15^ ^]^ A recent study identified TASL as an essential adaptor protein that mediates IRF5 activation downstream of TLR7–9 signaling, functionally analogous to the IRF3 adaptors STING, MAVS, and TRIF.^[^ ^16^ ^]^ SLC15A4 has been reported to recruit TASL to the endolysosome, a prerequisite for IRF5 activation.^[^ ^16^, ^17^, ^18^, ^19^ ^]^ However, the regulatory mechanisms that govern the TLR7–TASL–IRF5 axis in psoriasis remain unclear.

In this study, we demonstrate that deletion of the m^6^A writer Mettl3 in mouse macrophages mitigates imiquimod (IMQ)‐induced psoriasis‐like inflammation and inhibits M1 polarization by reducing m^6^A levels and destabilizing *Slc15a3* mRNA. Conversely, deletion of the m^6^A eraser Alkbh5 produces the opposite effect. Mechanistically, we show that *SLC15A3* promotes the recruitment of TASL to endolysosomes upon TLR7 stimulation, thereby facilitating IRF5 activation and amplifying downstream inflammatory signaling.

## 2 Results

### 2.1 Conditional Knockout of *Mettl3* in Macrophages Alleviates Psoriasis‐Like Phenotype in Mice

**Figure 1:**
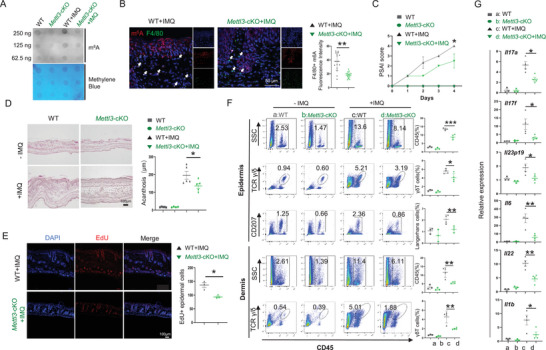
*Mettl3* deficiency in macrophages alleviates psoriasis‐like phenotype in mice. A) Dot blot of m^6^A (250, 125, or 62.5 ng total RNA) in the BMDMs from *Mettl3^f/f^;Lyz2‐Cre^±^*  and *Mettl3^f/f^* mice. B) m^6^A (red) and F4/80 (green) co‐immunofluorescence staining in IMQ‐induced lesions of WT and *Mettl3* cKO mice (left panel), and statistical analysis of m^6^A fluorescence intensity in F4/80^+^ cells (right panel). Arrows indicate F4/80^+^ macrophages. Scale bar, 50 µm. Each dot represents one cell, 3 mice for each group. C) Psoriasis area and severity index (PASI score) in IMQ‐induced lesions of WT and *Mettl3* cKO mice (*n* = 3). D) H&E staining of non‐lesions and IMQ‐induced psoriasis‐like lesions of WT and *Mettl3* cKO mice (*n* = 5). Left, representative picture; right, statistics of epidermal thickness. Scale bar, 100 µm. E) EdU (red) staining of IMQ‐induced psoriasis‐like lesions (*n* = 4) of WT and *Mettl3* cKO mice. Statistical analysis of the numbers of EdU^+^ cells in epidermis. Scale bar, 100 µm. F) Flow cytometry of immune cell infiltration, including CD45^+^ immune cells, γδT cells, and Langerhans cells (non‐lesion n = 3, lesion n = 4). The statistical data are shown in the right panels. G) The RNA expression of cytokines was quantified by qPCR in non‐lesions (*n* = 3) and IMQ‐induced psoriasis‐like lesions (*n* = 4) of WT and *Mettl3* cKO mice. Each dot represents one mouse. An unpaired *t* test was used for statistical analysis. ^*^ *P* < 0.01, ^**^ *P* < 0.01, ^***^ *P* < 0.001.

To investigate the role of N6‐methyladenosine (m^6^A) in psoriatic pathogenesis, we initially utilized *Alkbh5* knockout (*Alkbh5* KO) mice within the IMQ‐induced model of psoriasis. The deficiency of *Alkbh5* resulted in an aggravated psoriatic phenotype, as evidenced by increased PASI scores and pronounced epidermal hyperplasia (Figure S1A,B, Supporting Information). The *Alkbh5* KO mice also displayed elevated levels of inflammatory cytokines, a characteristic of psoriasis (Figure S1C, Supporting Information). To delineate the specific impact of m^6^A in macrophages, we engineered a conditional knockout of *Mettl3* (*Mettl3* cKO) within the *Lyz2*‐Cre mouse lineage (*Mettl3* ^loxp/loxp^; *Lyz2*‐Cre^+/−^). Dot blot experiments confirmed reduction of m^6^A modification in *Mettl3* KO bone marrow‐derived macrophages (BMDMs) (**Figure** **1**A). Consistently, a significant decrease in m^6^A was observed in F4/80^+^ macrophages at the IMQ‐induced psoriatic‐like lesion from *Mettl3* cKO mice (Figure 1B). We then assessed the function of *Mettl3* cKO in psoriasis. A significant reduction in the psoriasis area and severity index (PASI) score of IMQ‐treated *Mettl3* cKO mice was observed (Figure 1C). H&E staining results showed reduced epidermal thickening and hyperkeratosis (Figure 1D). EdU staining demonstrated suppressed proliferation of epidermal cells (Figure 1E). The data of flow cytometry revealed decreased infiltration of epidermal CD45^+^ cells, Langerhans cells, and γδ T cells, as well as dermal CD45^+^ and γδ T cells in the psoriatic‐like lesions of *Mettl3* cKO mice (Figure 1F; please refer to Figure S2, Supporting Information for gating strategy). Quantitative RT‐PCR analyses showed decreased expression of inflammatory cytokines, including *Il17a*, *Il17f*, *Il23p19*, *Il6*, *Il22*, and *Il1b*, in the skin lesions of *Mettl3*‐cKO mice (Figure 1G). Collectively, these data suggested that knockout of *Mettl3* in macrophages alleviated psoriasis‐like phenotype in mice.

### 2.2 Conditional Knockout *Alkbh5* Exacerbates Psoriasis‐Like Phenotype in Mice

**Figure 2:**
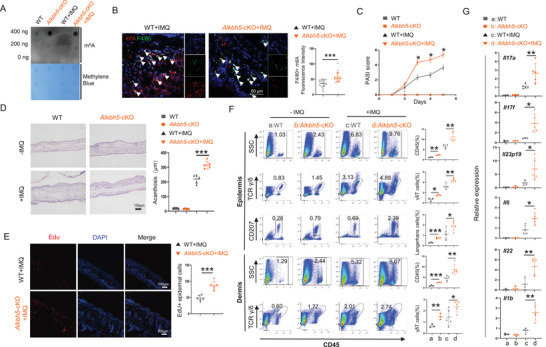
*Alkbh5* deficiency in macrophages exacerbates psoriasis‐like phenotype in mice. A) Dot blot of m^6^A (250, 125, or 62.5 ng total RNA) in the BMDMs from *Alkbh5^f/f^;Lyz2‐Cre^±^*  and *Alkbh5^f/f^* mice. B) m^6^A (red) and F4/80 (green) co‐immunofluorescence staining in IMQ‐induced lesions of WT and *Alkbh5* cKO mice (left panel), and statistical analysis of m^6^A fluorescence intensity in F4/80^+^ cells (right panel). Arrows indicate F4/80^+^ macrophages. Scale bar, 50 µm. Each dot represents one cell, 3 mice for each group. C) Psoriasis area and severity index (PASI score) in IMQ‐induced lesions of WT and *Alkbh5* cKO mice (*n* = 3). D) H&E staining of non‐lesions (*n* = 3) and IMQ‐induced psoriasis‐like lesions (*n* = 5) of WT and *Alkbh5* cKO mice. Left, representative picture; right, statistics of epidermal thickness. Scale bar, 100 µm. E) EdU (red) staining of IMQ‐induced psoriasis‐like lesions of WT and *Alkbh5* cKO mice (*n* = 5). Statistical analysis of the numbers of EdU^+^ cells in epidermis. Scale bar, 100 µm. (F) Flow cytometry of immune cell infiltration, including CD45^+^ immune cells, γδT cells, and Langerhans cells (non‐lesion n = 3, lesion n = 4). The statistical data are shown in the right panels. G) The RNA expression of cytokines was quantified by qPCR in non‐lesions (*n* = 3) and IMQ‐induced psoriasis‐like lesions (*n* = 5) of WT and *Alkbh5* cKO mice. Each dot represents one mouse. An unpaired *t* test was used for statistical analysis. ^*^ *P* < 0.01, ^**^ *P* < 0.01, ^***^ *P* < 0.001.

We next examined the IMQ‐induced phenotype in conditional knockout mice lacking the m^6^A demethylase *Alkbh5* (*Alkbh5* ^loxp/loxp^; *Lyz2*‐Cre^+/−^). Similarly, an increase of m^6^A modification in *Alkbh5* KO BMDMs was revealed by Dot blot (**Figure** **2**A), as well as a significant elevation was observed in F4/80^+^ macrophages in the IMQ‐induced psoriatic‐like lesions from *Alkbh5* cKO mice (Figure 2B). PASI scoring, H&E staining, and EdU incorporation assays demonstrated disruption of *Alkbh5* exacerbated psoriatic‐like phenotypes (Figure 2C–E). FACS and qRT‐PCR assays showed that the infiltration of immune cells into the skin lesions and inflammatory cytokine expression was significantly increased (Figure 2F,G). These findings indicated that *Alkbh5* deletion exacerbated the IMQ‐induced psoriasis phenotype, which was consistent with the observations in the *Mettl3* cKO mice.

### 2.3 The N6‐Methyladenosine Modification in Macrophages Promotes M1 Polarization

**Figure 3:**
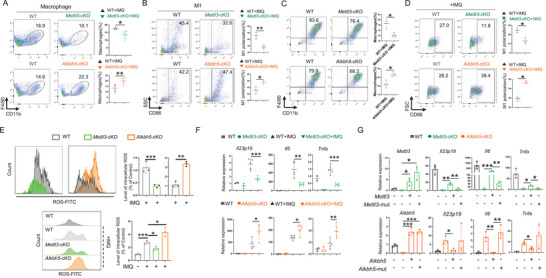
The N6‐methyladenosine in macrophages promotes M1 polarization. In vivo, IMQ‐induced psoriatic lesions were enzymatically digested into single‐cell suspensions, followed by flow cytometric analysis. In vitro, bone marrow‐derived macrophages (BMDMs) were isolated and subsequently stimulated with IMQ (2ug ml^−1^). A) The proportion of macrophages in IMQ‐induced psoriasis‐like lesions in mice (Up n = 4, down n = 6). The statistical data are shown in the right panels. (B) Proportion of M1 macrophages in IMQ‐induced psoriasis‐like lesions in mice (*n* = 3). The statistical data are shown in the right panels. C) Flow cytometry examines the percentages of M‐CSF induced macrophages from WT, *Alkbh5* cKO and *Mettl3* cKO bone marrow‐derived monocytes (*n* = 5). The statistical data are shown in the right panels. D) Flow cytometry examines the percentages of M1 macrophages from WT, *Alkbh5* cKO, and *Mettl3* cKO mice (*n* = 3). The statistical data are shown in the right panels. E) Flow cytometric examines reactive oxygen species (ROS) in macrophages from psoriatic skin lesions and in IMQ‐stimulated bone marrow‐derived macrophages (BMDMs) of WT, *Alkbh5* cKO, and *Mettl3* cKO mice (*n* = 3). The statistical analysis was shown in the right panels. Each dot represents one sample. F) The RNA expression of cytokines *Il23p19*, *Il6* and *Tnfα* are quantified by qPCR in WT, *Alkbh5* cKO, and *Mettl3* cKO BMDMs (n_IMQ‐_ = 3, n_IMQ+_ = 5). G) *Mettl3/Alkbh5* rescues the expression of *Il23p19*, *Il6*, and *Tnfa*. The wild‐types and catalytic‐activity‐dead mutants (*Mettl3* ^D395A&W398A^, *Alkbh5* ^H205A^) of Mettl3/Alkbh5 were delivered with lentiviral vectors into *Mettl3*‐KO and *Alkbh5*‐KO BMDMs, respectively (*n* = 3). The mRNA of *Mettl3, Alkbh5*, *Il23p19*, *Il6*, and *Tnfa* was examined by qRT‐PCR. Each dot represents one mouse. An unpaired *t* test was used for statistical analysis. ^*^ *P* < 0.01, ^**^ *P* < 0.01, ^***^ *P* < 0.001.

As the m^6^A modification in macrophages has been demonstrated to play a critical role in the IMQ‐induced psoriasis phenotype, we next examined the effect of this modification on the macrophage function. As shown in **Figure** **3**A, fewer macrophages infiltrated into the psoriatic lesions in *Mettl3* cKO mice than in WT mice, while more macrophages were observed in *Alkbh5* cKO mice. Macrophage M1 polarization is an essential biological event during the progression of psoriasis.^[^ ^7^, ^8^, ^15^, ^20^ ^]^ Then, we analyzed the proportion of M1 macrophages in the skin lesions from WT, *Mettl3*, and *Alkbh5‐*deficient mice using flow cytometry. Consistent with the phenotype observed in Figures 1 and 2, *Mettl3* cKO mice exhibited a decreased percentage of M1 macrophages among all macrophages, whereas *Alkbh5* cKO mice showed an increase in M1 macrophages (Figure 3B). Next, we used the in vitro differentiation and polarization of bone marrow‐derived monocytes (precursors of both tissue macrophages and dendritic cells) as models to test the impact of m^6^A modification on the development of macrophages. *Mettl3* deficiency leads to fewer macrophages differentiated from the monocyte precursors induced by macrophage colony‐stimulating factor (M‐CSF), and *Alkbh5* deficiency leads to more (Figure 3C). For in vitro M1 macrophage polarization, we used the TLR7 ligand IMQ to stimulate M‐CSF‐induced BMDMs and found that *Mettl3* deficiency attenuated the IMQ‐induced M1 macrophage polarization, whereas *Alkbh5* deficiency promoted it (Figure 3D). Previous studies have demonstrated a significant upregulation of reactive oxygen species (ROS) in M1‐polarized macrophages.^[^ ^21^, ^22^, ^23^ ^]^ Here, we assessed ROS levels in macrophages from murine psoriasis‐like skin lesions and BMDMs using flow cytometry. The results showed that knockout of *Mettl3* suppressed ROS production in macrophages, whereas knockout of *Alkbh5* enhanced ROS levels (Figure 3E). Consistently, *Mettl3* deficiency reduced the expression of pro‐inflammatory cytokines triggered by TLR7 signaling, including *Il23p19*, *Il6*, and *Tnfa*, whereas *Alkbh5* deficiency displayed an up‐regulation effect (Figure 3F). To verify that the phenotypes were indeed caused by *Mettl3* and *Alkbh5* deficiency, we performed rescue experiments with WT enzymes and catalytic activity‐dead mutants. As shown in Figure 3G, WT *Mettl3* and *Alkbh5* rescued the expression of *Il23p19*, *Il6*, and *Tnfa*, whereas the *Mettl3^D395A, W398A,^* and *Alkbh5 ^H205A^* mutants showed no effect, supporting that *Mettl3* and *Alkbh5* were responsible for the observed phenotype changes via an m^6^A‐dependent manner.

### 2.4 *Slc15a3* is Modified by m^6^A in Macrophages

**Figure 4:**
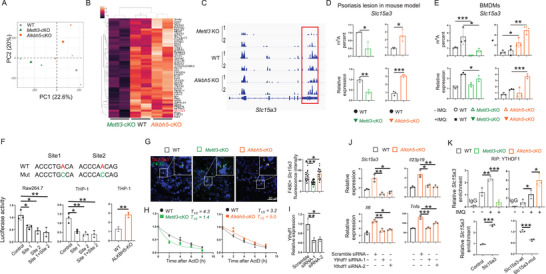
*Slc15a3* is modified by m^6^A in macrophages. A) Principal component analysis of the transcriptomes of BMDMs isolated from WT group (gray), *Alkbh5* cKO group (orange), and *Mettl3* cKO group (green) with 2ug ml^−1^ IMQ treatment 4 h (*n* = 2). B) Heatmap of genes with differential m^6^A peaks based on MeRIP‐seq data. C) Read density of m^6^A in *Slc15a3* transcript in WT, *Alkbh5* cKO, and *Mettl3* cKO BMDMs. D,E) qPCR and MeRIP‐qPCR analysis showed *Slc15a3* RNA level and m^6^A enrichment in WT, *Alkbh5* cKO and *Mettl3* cKO macrophages in psoriatic lesions (D) and BMDMs (E), respectively. F) Insertion of the identified *Slc15a3* m^6^A sites, but not its mutated versions, reduces the activity of a luciferase reporter in Raw264.7 and THP‐1 cells (*n* = 3). G) Co‐Immunoflurosecence staining of Slc15a3 (red) and F4/80^+^ (green). Arrows indicate F4/80^+^ macrophages. Each dot represents one cell, 3 mice for each group. Scale bar, 50 µm. The statistical data are shown in the right panel. H) Decay examination of *Slc15a3* mRNA (*n* = 3). Three group BMDMs were treated with Actinomycin D. I,J) *Ythdf1* knockdown reduces the expression of *Slc15a3*, *Il23p19*, *Il6*, and *Tnfa* in BMDMs with IMQ treatment. Each dot represents one mouse. K) (Upper panel) RNA immunoprecipitation (RIP)‐qPCR analysis of *Slc15a3* mRNA enrichment in YTHDF1‐IP fractions from Raw264.7 macrophages transfected with wild‐type *Slc15a3* (WT) or its m^6^A site mutant (A‐to‐C). (Lower panel) RIP‐qPCR analysis of *Slc15a3* mRNA in YTHDF1‐IP fractions from WT, *Mettl3*‐KO, and *Alkbh5* KO bone BMDMs treated with IMQ (2 µg/mL, 4 h). Data are normalized to input RNA levels and presented as fold change relative to WT (mean ± SEM, *n* = 3). An unpaired *t* test was used for statistical analysis. ^*^ *P* < 0.01, ^**^ *P* < 0.01, ^***^ *P* < 0.001.

To explore the molecular mechanisms by which m^6^A regulates macrophage function, we performed MeRIP‐seq on BMDMs from WT, *Mettl3* cKO, and *Alkbh5* cKO mice. Quality control analysis of the sequencing data indicated that m^6^A peaks were predominantly enriched around the stop codon (Figure S3A, Supporting Information), and principal component analysis (PCA) showed distinct compositional differences among the three groups (**Figure** **4**A). We performed motif search among the m^6^A regions and discovered GGACU as the most frequent motif, consistent with the reported RRACH motif for m^6^A modification (Figure S3B, Supporting Information). Interestingly, genes with differential m^6^A modification in *Mettl3* cKO and *Alkbh5* cKO relative to WT were enriched in psoriasis‐related pathways, including JAK‐STAT signaling pathway, Th17 cell differentiation, and Interleukin‐1 beta production (Figure S3C,D, Supporting Information). We then clustered genes whose m^6^A modification was decreased in *Mettl3* cKO and increased in *Alkbh5* cKO cells (Figure 4B). Based on the reported functions in the literatures, we selected *RelB*, *Slc15a3*, *Gpnmb*, *Arhgap25*, *RhoB*, *Cd274*, and *Cd22* to test their mRNA expression in BMDMs and found that *RelB* and *Slc15a3* showed decreased mRNA expression in *Mettl3* deficient cells and increased mRNA expression in *Alkbh5* deficient cells (Figure S4A, Supporting Information), indicating a regulatory role of m^6^A modification on mRNA expression. Since the m^6^A modification of *RelB*, a subunit of NF‐κB known to regulate immune responses,^[^ ^24^, ^25^, ^26^ ^]^ remained unchanged (Figure S4C, Supporting Information), we focused our further investigation on ***Slc15a3***, a less‐studied gene in immune regulation. MeRIP‐seq data revealed that the m^6^A modification sites were located within the 3’‐UTR region of *Slc15a3* (Figure 4C). To test whether m^6^A modification of *Slc15a3* occurred in the macrophages within the IMQ‐induced psoriasis‐like lesion, we sorted out macrophages to perform MeRIP‐qPCR and qRT‐PCR assays and found that the 3’‐UTR region, not 5’‐UTR region or exon 2, was m^6^A modified and that the modification level was positively correlated to mRNA expression level (Figure 4D; Figure S4B, Supporting Information). In addition, consistent results were observed in the BMDMs from WT, *Mettl3* cKO, and *Alkbh5* cKO mice treated with or without IMQ (Figure 4E). Moreover, using a luciferase reporter assay, we found that the identified two m^6^A sites at the 3’‐UTR region of *Slc15a3* mRNA conferred transcript destabilization in Raw 264.7 and THP‐1 cells in a manner dependent on the integrity of the m^6^A site, as an A‐to‐C mutation nullified this effect (Figure 4F). Last, immunofluorescence staining showed a reduced expression of SLC15A3 protein in F4/80^+^ macrophages in *Mettl3* cKO lesion, and an increased expression in *Alkbh5* cKO lesion (Figure 4G).

**Figure 5:**
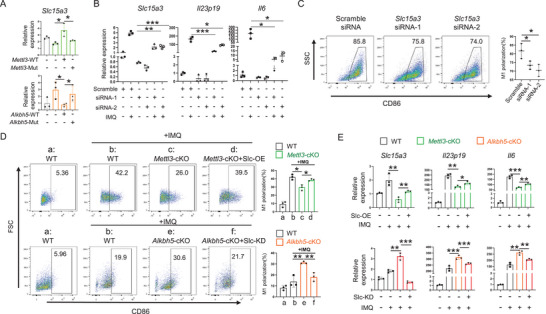
Slc15a3 is a key mediator for M1 polarization in *Mettl3* and *Alkbh5* deficient macrophages. A) Mettl3/Alkbh5 rescues the expression of *Slc15a3*. The wild‐types and catalytic‐activity‐dead mutants (*Mettl3* ^D395A&W398A^, *Alkbh5* ^H205A^) of *Mettl3/Alkbh5* were delivered with lentivirus vectors in *Mettl3*‐KO and *Alkbh5*‐KO BMDMs, respectively. Each dot represents one mouse (*n* = 3). The mRNA of *Slc15a3* was examined by qRT‐PCR. B,C) Slc15a3 mediates IMQ‐induced expression of *Il23p19*, *Il6*, and CD86. Two independent siRNAs targeting *Slc15a3* were transfected into Raw264.7 macrophages, and the mRNA expression levels of *Il23p19* and *Il6* were examined by qRT‐PCR (B), and the cell membrane expression of CD86 was examined by FACS (C). *n* = 3. Slc15a3 mediates M1 polarization. D,E) Slc15a3 functions downstream of Mettl3 and Alkbh5 to mediate IMQ‐induced M1 polarization*. Mettl3* KO BMDMs were infected with lentiviral vector encoding *Slc15a3* and *Alkbh5* KO BMDMs were transfected with *Slc15a3* specific siRNA, The proportion of M1 macrophages, expressing the CD86 marker on the cell surface (D) and the mRNA expression of *Slc15a3*, *Il23p19* and *Il1b* (E) were detected by flow cytometry and qRT‐PCR in WT, WT+IMQ, *Mettl3* KO‐*Slc15a3* OE +IMQ, *Alkbh5* KO‐*Slc15a3* KD+IMQ groups (*n* = 3). An unpaired *t* test was used for statistical analysis. ^*^ *P* < 0.01, ^**^ *P* < 0.01, ^***^ *P* < 0.001.

Based on the positive correlation between m^6^A modification and mRNA expression, we hypothesized that m^6^A modification stabilized *Slc15a3* mRNA. To test this idea, RNA synthesis was inhibited with actinomycin D, and the RNA degradation rate was examined by qRT‐PCR. The data showed that the stability of *Slc15a3* mRNA was reduced in *Mettl3* cKO compared to WT (Half‐life, 1.4 h VS 4.2 h), and increased in *Alkbh5* cKO (Half‐life, 5.0 h VS 3.2 h) (Figure 4H). We next tried to identify the m^6^A reader responsible for stabilizing *Slc15a3* mRNA. YTHDF1^[^ ^27^, ^29^, ^30^, ^31^, ^32^, ^33^ ^]^ have been reported to stabilize m^6^A modified mRNA. The results of single‐cell RNA sequencing in the lesions of psoriasis patients and RNA sequencing in BMDMs revealed that YTHDF1 and IGF2BP2 were expressed in macrophages (Figure S5A,B, Supporting Information). Knockdown of *Ythdf1* with two independent siRNAs rescued increased expression of *Slc15a3* as well as M1 inflammatory cytokines (*Il23p19*, *Il6*, and *Tnfa*) in *Alkbh5* cKO BMDMs (Figure 4I,J). However, knockdown of IGF2BP2 had no effect (Figure 5C,D, Supporting Information). Further, we performed RNA immunoprecipitation (RIP) assays using a YTHDF1‐specific antibody. As shown in Figure 4K, in *Mettl3* knockout BMDMs, the binding between YTHDF1 and *Slc15a3* mRNA was decreased, and on the contrary, in *Alkbh5* knockout BMDMs, the binding was increased (Figure 4K, upper panel). In Raw264.7 macrophages transfected with wild‐type *Slc15a3*, YTHDF1 antibody robustly enriched *Slc15a3* mRNA, whereas mutation of the m^6^A consensus motifs significantly decreased this interaction, confirming the m^6^A‐dependent binding of YTHDF1 to *Slc15a3* transcripts (Figure 4K, down panel).

Collectively, all the above data demonstrated that the m^6^A writer Mettl3, eraser Alkbh5, and reader Ythdf1 regulated m^6^A modification in the 3’‐UTR region of *Slc15a3* mRNA for stabilization.

### 2.5 SLC15A3 is a Key Mediator for M1 Polarization in *mettl3* and *alkbh5* Deficient Macrophages

We next investigated the role of SLC15A3 in macrophage M1 polarization. In rescue experiments using BMDMs, we confirmed that *Mettl3* and *Alkbh5* positively and negatively regulate *Slc15a3* mRNA expression, respectively, in an m⁶A‐dependent manner. Wild‐type (WT) *Mettl3* and *Alkbh5*, but not their catalytically inactive mutants, were able to restore *Slc15a3* expression (**Figure** **5**A). In Raw264.7 macrophages, knock‐down of *Slc15a3* with two independent siRNAs down‐regulated IMQ‐induced mRNA expression of pro‐inflammatory cytokines *Il23p19* and *Il6*, as well as the surface expression of the M1 marker CD86, indicating that SLC15A3 is required for IMQ‐induced M1 polarization (Figure 5B,C). To further assess whether SLC15A3 mediates the effects of m^6^A modification on polarization, we overexpressed *Slc15a3* in primary *Mettl3*‐deficient BMDMs using a lentiviral vector and knocked down *Slc15a3* in *Alkbh5*‐deficient BMDMs using siRNA‐2. Overexpression of *Slc15a3* in *Mettl3* cKO macrophages increased CD86 surface expression and upregulated *Il23p19* and *Il6* expression. Conversely, knockdown of *Slc15a3* in *Alkbh5* cKO macrophages reduced CD86 expression and cytokine levels (Figure 5D,E).

### 2.6 SLC15A3 Promotes Lysosomal Localization of TASL to Activate TASL‐IRF5 Signal

**Figure 6:**
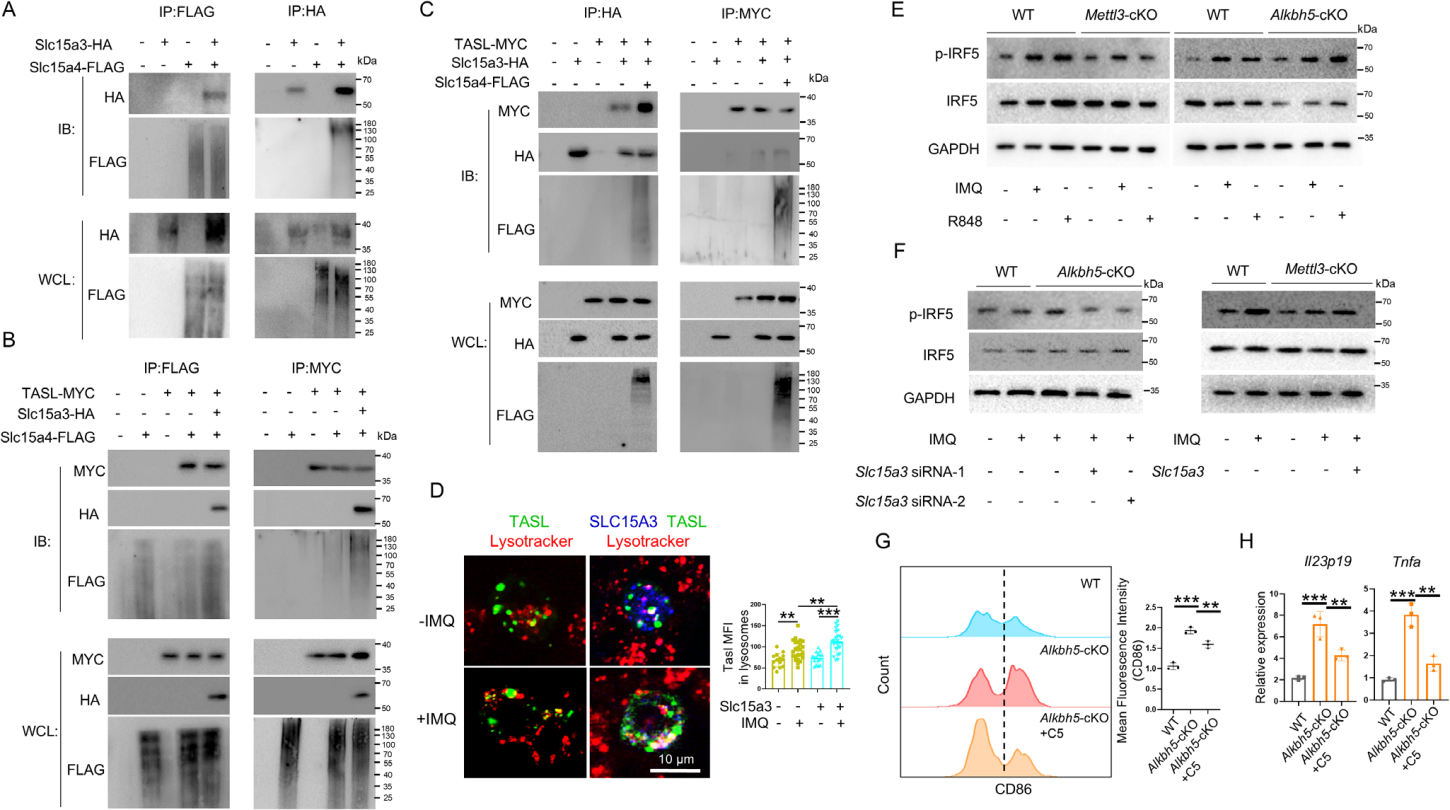
Slc15a3 promotes endolysosomal localization of TASL to activate TASL‐IRF5 signal. A–C) Protein‐protein interactions between Slc15a3, Slc15a4, and TASL. Co‐Immunoprecipitation assays were performed with cell extracts from the HEK239T cells transfected with the plasmids encoding the indicated genes. D) Slc15a3 promotes endolysosomal localization of TASL. The plasmids encoding *Slc15a3*‐BFP (blue) and *Tasl*‐Aausfp1 (green) were transfected into Raw264.7 macrophages. Lysotracker (red) was used for endolysosome labeling. Each dot represents one lysosome. Scale bar, 10 µm. E) m^6^A modification mediates IMQ‐ or R848‐stimulated IFR5 activation. Western blot showed the expression of phosphorylated IRF5 (p‐IRF5), total IRF5, and GAPDH in WT, *Alkbh5* KO, and *Mettl3* KO BMDMs treated with TLR7 ligands IMQ or R848. F) Slc15a3 functions downstream of m^6^A modification to mediate IMQ‐stimulated IRF5 activation. Western blot showed the expression of p‐IRF5, IRF5, and GAPDH in WT, *Alkbh5* KO, and *Mettl3* KO BMDMs transfected with the siRNA targeting *Slc15a3* or the plasmid encoding *Slc15a3*. G,H) Flow cytometry of IMQ induced M1 polarization and the mRNA expression of *Il23p19* and *Tnfa* in WT, *Mettl3*‐KO, and *Alkbh5*‐KO BMDMs. C5 was employed to inhibit IRF5 signaling activated by TASL. Flow cytometry was used to assess the proportion of CD86⁺ macrophages in IMQ‐stimulated bone marrow‐derived macrophages (BMDMs) with C5 treated 24 h (G). In parallel, quantitative PCR (qPCR) was performed to evaluate the expression levels of Il23 and Tnfa following IMQ stimulation (H). The data represent one out of three independent biological replicates. ^*^ *P* < 0.01, ^**^ *P* < 0.01, ^***^ *P* < 0.001.

Recent studies showed that TASL is an innate immune endolysosomal adaptor for TLR7‐9 signaling, revealing a clear mechanistic analogy with the IRF3 adaptors STING, MAVS, and TRIF, and SLC15A4 is responsible for recruiting TASL to the endolysosome.^[^ ^16^, ^17^, ^18^, ^19^, ^34^ ^]^ In addition, SLC15A3 and SLC15A4 have been reported to interact with each other to regulate immune response.^[^ ^35^ ^]^ SLC15A3 is localized on the endolysosomal membrane of macrophages and contributes to inflammation.^[^ ^36^, ^37^ ^]^ Therefore, we hypothesized that SLC15A3 may regulate the SLC15A4–TASL complex, thereby influencing downstream IRF5 activation. To test this hypothesis, we co‐transfected HEK293T cells with plasmids encoding SLC15A3‐HA, SLC15A4‐FLAG, and TASL‐MYC, followed by co‐immunoprecipitation (co‐IP) assays. As reported previously, SLC15A4 appeared glycosylated and migrated as multiple high‐molecular‐weight bands.^[^ ^16^, ^19^ ^]^ We confirmed interactions between SLC15A3 and SLC15A4, as well as between SLC15A4 and TASL (**Figure** **6**A,B). Notably, we also observed direct interaction between SLC15A3 and TASL, and found that SLC15A3 enhanced the interaction between SLC15A4 and TASL, and vice versa (Figure 6B,C), suggesting that these three proteins may form a functional complex. As the recruitment of TASL by SLC15A4 to the endolysosome is a crucial event for TLR7‐IRF5 signaling,^[^ ^16^ ^]^ we checked the impact of Slc15a3 on the subcellular localization of TASL in macrophages. Immunofluorescence staining revealed that endogenous SLC15A3 was localized on lysosomes (Figure S6A, Supporting Information). Live imaging of fluorescent proteins and Lysotracker (red) in Raw264.7 macrophages showed that TASL‐Aausfp1 (green) was localized in lysosome, IMQ treatment induced more TASL recruited to lysosome, and overexpression of SLC15A3 (SLC15A3‐BFP, blue) further enhanced the lysosomal localization of TASL (Figure 6D). Co‐transfection of SLC15A3‐BFP, SLC15A4‐smURFP (purple), and TASL‐Aausfp1 confirmed the co‐localization of all three proteins at lysosomes, and that SLC15A3 facilitates TASL recruitment upon IMQ stimulation (Figure S6B, Supporting Information). Next, we assessed whether m^6^A modification activated IRF5 signaling pathway and whether this activation process was dependent on SLC15A3. The results showed that in BMDMs, *Mettl3* knockout inhibited IRF5 activation, whereas *Alkbh5* knockout enhanced IRF5 activation (Figure 6E). Overexpression of SLC15A3 in *Mettl3*‐cKO BMDMs enhanced IRF5 activation, while knockdown of SLC15A3 in *Alkbh5* cKO BMDMs suppressed IRF5 activation (Figure 6F). Meanwhile, *Mettl3* and *Alkbh5* showed no obvious effect on the activation of IKKβ and JNK (Figure S6C, Supporting Information). And a recent study has demonstrated that C5 inhibits IRF5 signaling by locking SLC15A4 in a TASL‐binding‐incompetent lysosomal outward‐open conformation.^[^ ^34^ ^]^ Here, the data showed that C5 suppressed M1 polarization and the expression of *Il23* and *Tnfa* in IMQ‐induced *Alkbh5*‐KO BMDMs (Figure 6G,H). All the above observations indicated that Slc15a3 promoted lysosomal localization of TASL to the activated downstream IRF5 signal.

### 2.7 The METTL3/ALKBH5‐m^6^A‐SLC15A3 Axis in Macrophages is Associated with the Severity of Psoriasis

**Figure 7:**
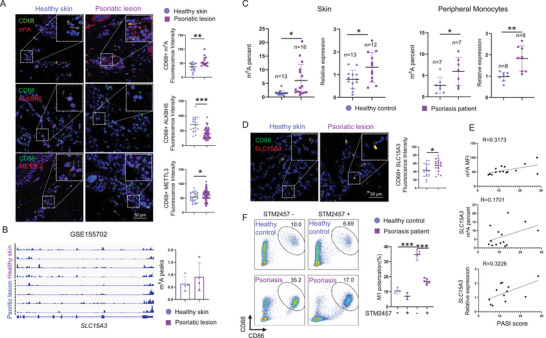
The METTL3/ALKBH5‐m^6^A‐SLC15A3 axis in macrophages is associated with the severity of psoriasis. A) Immunofluorescence staining shows the expression levels of m^6^A/ALKBH5/METTL3 (red) in CD68^+^ macrophages (green) of healthy skin and psoriatic lesions. Each dot represents one cell, 3 mice for each group. Scale bar, 50 µm. The corresponding fluorescence intensity statistical analysis is shown in the right panels. Each dot represents one microscope field of view from 6 healthy people and 8 psoriasis patients. An unpaired *t* test was used for statistical analysis. B) IGV visualization shows the m^6^A peaks located on *SLC15A3* transcripts in healthy skin and psoriatic lesions (data analysis based on the public database, GSE155702). (C) MeRIP‐qPCR and qRT‐PCR reveal the m^6^A modification at the 3’‐UTR and the mRNA expression levels of *SLC15A3* in the skin and peripheral monocytes from healthy people and psoriasis patients. D) Immunofluorescence staining shows the expression levels of SLC15A3 (red) in CD68^+^ macrophages (green) of healthy skin and psoriatic lesions. The experiments were performed as the same as those in A. E) Pearson correlation analysis shows the positive correlation between the PASI scores and m^6^A Mean Fluorescence Intensity (MFI), m^6^A modification of *SLC15A3*, and the relative expression of *SLC15A3* in CD68^+^ macrophages. Each dot represents one clinical sample. F) The proportion of M1‐polarized macrophages. Peripheral blood mononuclear cells (PBMCs) were isolated from healthy donors (*n* = 3) and psoriasis patients (*n* = 8), followed by monocyte separation and differentiation into macrophages. During IMQ‐induced M1 polarization, macrophages were treated with the METTL3 inhibitor STM2457. The statistical analysis was shown in the right panels. Each dot represents one sample. ^*^ *P* < 0.01, ^**^ *P* < 0.01, ^***^ *P* < 0.001.

We next investigated the clinical relevance of the METTL3/ALKBH5‐m^6^A‐SLC15A3 axis in psoriasis. Immunofluorescence staining revealed an increase in m^6^A modification in CD68^+^ macrophages within psoriatic lesions, accompanied by a down‐regulation of ALKBH5 protein and an up‐regulation of METTL3 protein (**Figure** **7**A). Further, we observed an increase in m^6^A modification and mRNA expression of SLC15A3 in both psoriatic lesions and CD14^+^ monocytes from peripheral blood (Figure 7B,C). Moreover, immunofluorescence staining results demonstrated an increased protein expression of SLC15A3 in CD68^+^ macrophages within psoriatic lesions (Figure 7D). Correlation analysis showed that m⁶A levels in CD68⁺ cells, m⁶A modification of *SLC15A3*, and *SLC15A3* mRNA levels were all positively correlated with psoriasis severity, as measured by the Psoriasis Area and Severity Index (PASI) (Figure 7E). These results indicated that increased m^6^A modification and thus enhanced stability of SLC15A3 mRNA and protein expression in macrophages might drive psoriasis pathogenesis.

STM2457, a selective inhibitor of METTL3 enzymatic activity, was used to treat macrophages derived from the peripheral blood of healthy donors and psoriasis patients. The results demonstrated that M1 polarization was significantly elevated in macrophages from psoriasis patients compared to those from healthy individuals. Notably, treatment with the METTL3 inhibitor markedly attenuated M1 polarization in psoriasis‐derived macrophages (Figure 7F). Meanwhile, STM2457 ameliorated the IMQ‐induced psoriasis‐like phenotype, as evidenced by reduced PASI scores, alleviation of histopathological hallmarks including epidermal hyperplasia, hyperkeratosis, and parakeratosis, as well as downregulated expression of pro‐inflammatory mediators such as *Il17a*, *Il17f*, *S100a8*, and *Tnfa* (Figure S7, Supporting Information).

## 3 Discussion

The role of N6‐methyladenosine (m^6^A) modification in regulating various physiological and pathological processes has been widely recognized.^[^ ^38^, ^39^ ^]^ However, the specific role of m^6^A in psoriasis remains underexplored. In this study, we provide a comprehensive investigation into the involvement of m^6^A RNA modification in macrophages and its contribution to the pathogenesis of psoriasis. Macrophages are pivotal in the development and persistence of psoriatic lesions by secreting pro‐inflammatory cytokines, including TNFα, IL‐23, and so on.^[^ ^2^, ^40^ ^]^ The increased presence of M1 macrophages within psoriatic lesions acts as a major driver of disease progression.^[^ ^7^ ^]^ Our findings highlight the role of m^6^A modification in regulating macrophage function in psoriasis, focusing on how m^6^A modification of SLC15A3 contributes to M1 macrophage polarization, thereby advancing our understanding of immune regulation in psoriasis. Additionally, we demonstrate that Slc15a3 impacts macrophage polarization through modulation of the TASL‐IRF5 signaling pathway.^[^ ^41^, ^42^ ^]^

Despite recognition of m^6^A's critical roles in various autoimmune diseases,^[^ ^43^, ^44^, ^45^, ^46^ ^]^ its function in psoriasis remains largely unexplored. A prior MeRIP‐seq study suggested that m^6^A modification may play a significant role in psoriatic lesions.^[^ ^47^ ^]^ However, this study focused on whole‐lesion analysis and did not investigate the underlying mechanisms of m^6^A modification in specific cell types, such as macrophages. In contrast, our research reveals increased m^6^A modification in psoriatic macrophages, with a positive correlation between m^6^A levels and PASI scores. Moreover, we observed upregulation of the m^6^A “writer” METTL3 and downregulation of the “eraser” ALKBH5 in psoriatic macrophages. These findings suggest that m^6^A modification in macrophages could represent a novel therapeutic target for psoriasis. Importantly, in vivo experiments demonstrate that reducing m^6^A modification in macrophages alleviates psoriasis symptoms, whereas enhancing m^6^A exacerbates disease severity. While m^6^A modification has also been implicated in other cell types involved in psoriasis, such as keratinocytes and T cells,^[^ ^48^, ^49^ ^]^ accumulating evidence suggests that m^6^A targets distinct downstream signaling pathways in a cell‐type‐specific manner. In keratinocytes, m^6^A influences cytokine production and lipid metabolism, whereas in T cells, it modulates IL‐17 expression and Th17 differentiation. In contrast, our study identifies Slc15a3 as a key m^6^A‐modified transcript in macrophages, acting through the TASL–IRF5 signaling axis to promote M1 polarization and inflammation. These findings underscore the layered complexity of m^6^A‐mediated regulation in psoriasis and highlight the need to consider cell‐specific m^6^A‐target networks when developing therapeutic strategies.

While m^6^A's regulatory impact on macrophage function has been reported, its effects are context‐dependent, varying under different physiological conditions.^[^ ^12^, ^13^, ^14^ ^]^ For example, m^6^A modification of *Socs1* suppresses macrophage inflammation during bacterial infections,^[^ ^14^ ^]^ whereas m^6^A modification of *Irakm* enhances macrophage activation in response to TLR4 ligands.^[^ ^13^ ^]^ These studies have primarily focused on the TLR4 signaling pathway in macrophages, but the role of m^6^A in regulating macrophage polarization through TLR7 signaling remains poorly understood. Activation of TLR7 has been shown to play a pivotal role in psoriasis pathogenesis.^[^ ^15^, ^50^, ^51^ ^]^ In the present study, we utilized *Mettl3* and *Alkbh5* knockout mice to investigate how m^6^A regulates macrophage functions in psoriasis. Our data reveal that m^6^A modification of Slc15a3 promotes M1 macrophage polarization via TLR7 signaling, with increased m^6^A modification of Slc15a3 observed in psoriatic lesions.

Slc15a3, a solute carrier protein, has been implicated in macrophage activation through the TLR7 pathway,^[^ ^37^ ^]^ although the precise molecular mechanisms remain unclear. A recent study has identified TASL as a key mediator linking endolysosomal TLRs to the IRF5 transcription factor via Slc15a4.^[^ ^16^ ^]^ Slc15a3 and Slc15a4, members of the same protein family with similar structures and cellular localization, exhibit inducible and constitutive expression patterns in macrophages, respectively.^[^ ^16^, ^19^, ^36^, ^37^, ^52^, ^53^ ^]^ In this study, we confirmed an interaction between Slc15a3 and the Slc15a4‐TASL complex using co‐immunoprecipitation experiments. Fluorescence microscopy further showed that Slc15a3 enhances the recruitment of TASL to lysosomes, thereby activating downstream IRF5. Our findings are consistent with previous reports,^[^ ^16^ ^]^ where the interaction between Slc15a3 and TASL was shown in HEK293T cells (Extended Data Figure 3e in the reference and Figure 6C in this study). Additionally, in Slc15a4 knockout CAL‐1 plasmacytoid dendritic cells, Slc15a3 failed to bind TASL (Extended Data Figure 8h in the reference), indicating that the interaction is dependent on Slc15a4. While our study underscores the importance of SLC15A3 in psoriasis, it remains unclear whether this interaction occurs similarly in other inflammatory diseases. Further studies will be necessary to assess the broader applicability of SLC15A3 in macrophages across diverse immune contexts.

In conclusion, our study establishes that m^6^A modification of Slc15a3 stabilizes its mRNA and enhances protein expression, thereby amplifying the TASL‐IRF5 signaling pathway, promoting M1 macrophage polarization, and contributing to the pathogenesis of psoriasis. These cellular and molecular insights provide a foundation for developing targeted therapeutic strategies for psoriasis, potentially guiding future treatments aimed at modulating m^6^A modification in immune cells.

## 4 Experimental Section

### Mice

*Alkbh5* ^fl/fl^ mice were provided by Dr. Xiong Cao from the National Key Laboratory of Neurobiology at Southern Medical University, Guangzhou, China. *Mettl3* ^fl/fl^; *Lyz2*‐Cre mice were purchased from the Cyagen Company. All mice were bred and housed under specific pathogen‐free conditions and used in experiments following the National Institutes of Health Guide for the Care and Use of Laboratory Animals, with approval from the Scientific Investigation Board of Southern Medical University (L2018162), Guangzhou, China.

### Cell Culture

Raw264.7, HEK293T, and THP1 cells were purchased from ATCC. Raw264.7 and HEK293T cells were cultured in Dulbecco's modified Eagle's medium (DMEM, Life Technologies), and THP1 cells were cultured in RPMI 1640 medium (Life Technologies). All growth media were supplemented with 2 mm L‐glutamine (Life Technologies), 100 U mL^−1^ penicillin, 100 µg mL^−1^ streptomycin (Life Technologies), and 10% fetal bovine serum (VISTECH). All the cell lines in this study were cultured in no more than 10 passages. Cells were incubated at 37 °C in 5% CO2.

### Bone Marrow‐Derived Macrophages (BMDMs) were Isolated from Mice

Mice were sacrificed by CO_2_ exposure, and the skin and tissues from the lower body and legs were removed. The femoral and pelvic bones were cleaned, and the marrow was harvested by flushing the bones with cell medium or PBS using a 26 ½ gauge needle attached to a 10 mL syringe, collecting the marrow into a 50 mL tube. The marrow was then centrifuged at 450g for 5 min at 4°C, and the supernatant was discarded. Red blood cells were lysed with RBC lysis buffer for 4 min at room temperature, followed by resuspension in cold cell medium or PBS. The cell suspension was filtered, centrifuged again, and the cells were counted. BMDMs were seeded at a concentration of 5 × 10⁶ cells per well in a 6‐well plate, with DMEM supplemented with 20% FBS and 30 ng/mL M‐CSF. The medium was replenished on days 3–4. On days 7–10, the cells were harvested for further experimentation.

### Animal Model of Psoriasis

Mice aged 8–12 weeks received daily topical application of 5% IMQ cream (Aldara, 3M Pharmaceuticals) on their shaved right ear or back for 3–5 consecutive days. The severity of the psoriatic phenotype was assessed using PASI scores, summing the severity of desquamation and erythema. For cell proliferation studies, mice were injected intraperitoneally with EdU (50 mg kg^−1^) and sacrificed 48 h later. For inhibitor studies, mice received daily topical application of STM2457 formulated in corn oil at a dose of 100 mm during the induction of the IMQ‐induced psoriasis‐like model. STM2457 was applied directly to the shaved dorsal skin at the site of IMQ treatment throughout the course of the experiment.

### Plasmid Construction, Cell Transfection, and Lentivirus Transduction

cDNAs for mouse Slc15a3, Slc15a4, TASL, Mettl3/Mettl3^D395A,W398A^, and Alkbh5/Alkbh5^H205A^ were synthesized (Genewiz, China) and used for cloning. pCAG‐TASL‐MYC‐2A‐mCherry‐pA, pCAG‐Slc15a3‐HA‐2A‐mCherry‐pA, pCAG‐Slc15a4‐FLAG‐2A‐mCherry‐pA, pCAG‐Slc15a4‐smURFP‐pA, pCAG‐Slc15a3‐BFP‐pA and pCAG‐TASL‐AausFP1‐pA were generated by inserting amplified DNA fragments into the pCAG‐puro‐pA vector. The above plasmids were transfected using the Lipofectamine 3000 reagent (Invitrogen) following the manufacturer's instructions. *Mettl3/Mettl3* ^D395A,W398A,^ *Alkbh5/Alkbh5* ^H205A^ and *Slc15a3* were cloned into the LV‐EF1A‐mNeonGreen‐WPRE vector by Gibson Assembly. Lentivirus was packaged in HEK293FT cells through co‐transfecting each of the over‐expression constructs with the packaging vectors (PsPAX2, pMD2.G) into HEK293FT cells. Cells were then incubated at 37 °C for 8 h and refreshed with full medium. Viral supernatant was harvested 48 and 72 h post‐transfection. The lentivirus‐containing supernatant was filtered through a 0.45 µm filter, and lentiviral particles were concentrated by using the lentivirus concentration solution(YEASEN 41101ES50) according to the manufacturer's instructions. Lentiviral particles were resuspended in PBS and immediately aliquoted for subsequent quantification and storage at −80 °C. After titrating the lentiviral particles, BMDMs were infected with a concentration of 1 × 10^6^ virus particles per µL.

### Flow Cytometry and Fluorescence Activated Cell Sorting

After euthanizing the mice, the IMQ‐induced psoriasis‐like skin lesions and non‐lesional skin tissues were incubated in 2.5 mg/ml dispase II (Roche, 04942078001) at 37°C for 1 hr to separate the epidermis and dermis. The epidermis and dermis pieces were digested separately in 1 mg/ml collagenase solution for 1.5 hrs at 37°C. Single‐cell suspensions were stained with fluorophore‐conjugated antibodies and analyzed on a BD LSRFortessa flow cytometer. The following antibodies were used: CD45‐FITC (Biolegend, 103108), TCR‐γ/δ‐BV510 (Biolegend, 118131), F4/80‐APC (eBioscience, 17‐4801‐82), anti‐CD207‐PE (Biolegend, 144204), CD11b‐PE (Biolegend, 101208), and CD86‐PE/Cy7 (Biolegend, 103108). The FACS data were analyzed with FlowJo software.

For sorting *Lyz2* ^+^ cells from IMQ‐induced psoriasis‐like lesions of *Mettl3*/*Alkbh5* ^fl/fl^; *Lyz2*‐Cre^+/−^; R26‐tdTomato and *Mettl3*/*Alkbh5* ^fl/+^; *Lyz2*‐Cre^+/−^; R26‐tdTomato mice (controls), single cell suspensions of skin were prepared as described above, and tdTomato‐positive cells were sorted by flow cytometry. For the isolation of CD14^+^ monocytes from human peripheral blood mononuclear cells (PBMCs), PBMCs were separated from the blood by density gradient centrifugation. APC‐conjugated anti‐CD14 antibody (Biolegend, 325607) was then used to isolate monocytes by FACS.

Intracellular reactive oxygen species (ROS) levels were assessed in macrophages isolated from IMQ‐induced psoriatic skin lesions and in bone marrow‐derived macrophages (BMDMs). Cells were incubated with 10 µM H₂DCFDA (2′,7′‐dichlorodihydrofluorescein diacetate) (Beyotime, China) in serum‐free medium at 37°C for 30 min in the dark. After incubation, cells were washed twice with PBS and immediately analyzed by flow cytometry. Fluorescence intensity was measured in the FITC channel, and data were analyzed using FlowJo software. Relative ROS levels were quantified based on mean fluorescence intensity (MFI) and compared between WT, *Mettl3* cKO, and *Alkbh5* cKO groups.

### Immunofluorescence

Skin tissues were sectioned into 10‐µm cryosections and fixed with paraformaldehyde. For staining, the cryosections were washed in PBS, incubated in blocking buffer (5% BSA, 0.01% Triton X‐100 in PBS) for 30 min at room temperature, then stained with the primary antibodies overnight at 4 °C. Secondary antibodies were applied at room temperature in the dark. Nuclei were counterstained with 4'6‐diamidino‐2‐phenylindole (DAPI, Invitrogen, D1306). The following antibodies were used, ALKBH5 (Abcam, ab195377), METTL3 (Abcam, ab195352), m^6^A (Synaptic Systems, 202111), F4/80(Abcam, ab60343), CD68 (Abcam, ab213363), SLC15A3 (Invitrogen, PA5‐66097), LAMP1 (Abcam, ab25630). LysoTracker Red (Yesen, 40739ES50) was a red fluorescently labeled endolysosomal probe with a maximum excitation/emission wavelength of 577/590 nm.

### EdU Incorporation Assay

EdU staining was used for detecting DNA synthesis in proliferating cells. EdU was intraperitoneally injected into mice 48 h before euthanasia. Skin tissues were sectioned into 10‐µm cryosections and fixed with paraformaldehyde. The cryosections were washed with PBS and then stained with EdU Cell Proliferation Kit (Beyotime, C0075S). Nuclei were counterstained with 4'6‐diamidino‐2‐phenylindole (DAPI, Invitrogen, D1306).

### Histology

The skin was harvested immediately after the mice were killed and fixed overnight in 4% formaldehyde made in PBS (pH 7.2) at 4°C. The tissue was then processed, embedded in paraffin wax, and cut into 6 µm sections. The sections were stained with hematoxylin and eosin (H&E) procedure, and visualized using a light microscope (Nikon, Eclipse 80i, Japan). Epidermal thickness (×100 magnification) was measured using ImageJ software using the following formula: thickness (µm) = area (µm^2^) / length (µm).

### RNA Extraction and qRT‐PCR

Total RNA was extracted using TRIzol reagent (Yeasen Biotech, 10606ES60) and reverse transcribed into cDNA using the Evo M‐MLV RT Kit with gDNA Clean for qPCR II (Accurate Biotechnology cat. AG11711). Real‐time quantitative PCR (qPCR) was conducted in a LightCycler 96 (Roche, Basel, Switzerland) using the SYBR Green Premix Pro Taq HS qPCR Kit (Accurate Biotechnology cat. AG11701). The relative expression levels of target genes were normalized to those of GAPDH and quantified using the 2^−ΔΔCt^ method. The sequences of the primers used for qPCR were presented in Table S1 (Supporting Information).

### Western Blot and Co‐IP

Total protein was extracted using ice‐cold RIPA lysis buffer, snap freezing, and mechanical shearing. Lysates were cleared by centrifugation at 13 000 rpm for 10 min at 4°C. Protein supernatants were separated on 10% SDS‐PAGE and then transferred to PVDF membranes. After blocking with 5% non‐fat dry milk for 1 hr at room temperature, the membranes were incubated first with the primary antibodies against HA (MBL, M180‐3), MYC (Abcam, ab9106), FLAG (Invitrogen, MA1‐91878), IRF5 (Abcam, 181553), phospho‐IRF5^Ser437^ (Signalway Antibody, 12688), SAPK/JNK (Cell Signaling Technology, 9252), phospho‐SAPK/JNK^Thr183/Tyr185^ (Cell Signaling Technology, 4668), IKKβ (Cell Signaling Technology, 8943), phospho‐IKKα/β^Ser176/180^ (Cell Signaling Technology, 2697), or GAPDH (Proteintech, 60004‐1‐Ig) overnight at 4 °C, and then with secondary antibody for 1 hr at room temperature. The signals were detected using Immobilon Western Chemiluminescent HRP Substrate (Millipore, WBKLS0500). For immunoprecipitation experiments of overexpressed proteins, the Co‐IP assay was performed using Pierce Crosslink Magnetic IP/Co‐IP Kit (Thermo Scientific, 88805).

### MeRIP‐seq

MeRIP‐seq was performed as previously described.^[^ ^54^ ^]^ Briefly, to perform MeRIP‐seq with RNA from BMDMs, polyA‐tailed RNA was enriched from at least 10 µg of total RNA using the DYNABEADS MRNA PURIFICATION Kit (Invitrogen, 61006) according to the manufacturer's instructions, followed by purification with VAHTS RNA Clean Beads (Vazyme, N412‐02) and elution in 10–20 µL of nuclease‐free water. Next, the eluted RNA was fragmented at 94°C for 30 seconds using RNA Fragmentation Reagents (New England Biolabs, E6150S) and purified using the ZYMO RNA Clean & Concentrator Kit (Zymo Research, R1016). Proceed with the m^6^A RNA immunoprecipitation (IP) according to the established protocol, ensuring optimal m^6^A antibody (Cell Signaling Technology, 56593S) binding and RNA recovery. Following IP, the RNA library was constructed using the NEBNext Ultra II Directional RNA Library Prep Kit (New England Biolabs, E7760S) and purified with VAHTS DNA Clean Beads (Vazyme, N411‐02). Finally, the library was resolved on a 2% agarose gel, aiming for an ideal library size of ≈250 bp.

### MeRIP‐qPCR

RNA isolated from skin biopsies was fragmented, incubated with anti‐m^6^A antibody, eluted, and reverse‐transcribed. qRT‐PCR was conducted using specific primers for target genes and controls. The sequences of the primers used for MeRIP‐qPCR was presented in Table S1 (Supporting Information).

### RIP‐qPCR

To determine the specific binding sites of YTHDF1 on Slc15a3 mRNA, RIP‐qPCR was performed using a RIP Kit (BersinBio, Bes5101) according to the manufacturer's instructions. For the endogenous YTHDF1‐RIP, RAW264.7 cells, which were treated with IMQ, were plated into a 10‐cm dish and allowed to grow for 48 h. Then, the cells were lysed, and the lysate was incubated with anti‐YTHDF1 antibody (Proteintech, 17479‐1‐AP) or with a rabbit IgG antibody conjugated to Protein A/G beads in 1 mL RIP buffer overnight at 4°C. The beads were then washed five times with wash buffer, and the RNA was collected by adding TRIzol to the beads. For the YTHDF1‐RIP of wild‐type or mutant Slc15a3 mRNA in RAW264.7 cells, the cells were co‐transfected with the indicated plasmids (mouse YTHDF1, WT Slc15a3 (NM_023044.2), and c.2233A>C Slc15a3) for 48 h. The cells were then lysed, and the cell lysates were incubated with rabbit anti‐YTHDF1 antibody or with a rabbit IgG antibody conjugated to Protein A/G beads in 1 ml RIP buffer overnight at 4°C. Then, the beads were washed five times with wash buffer, and the RNA was collected by adding TRIzol to the beads. Finally, the input samples (10%) and all the IP RNA samples were subjected to RT–qPCR. The relative RIP enrichment was determined by calculating the 2^−Δ^ *^C^* ^t^ of the RIP sample relative to the input sample. The sequences of the plasmids used for RIP‐qPCR was presented in Table S1 (Supporting Information).

### m^6^A Dot Blot

Total RNA was extracted using TRIzol reagent, followed by quantification of RNA concentration. RNA was serially diluted to final concentrations in nuclease‐free water to ensure uniform loading for dot blot analysis. Incubated the serially diluted RNA at 95°C in a heat block for 3 mins to disrupt secondary structures and then immediately chilled the tubes on ice to prevent the re‐formation of secondary structures of RNA. A total of 2 µL of RNA was spotted onto a positively charged nylon membrane (Amersham RPN303B, GE) using a micropipette. The membrane was air‐dried briefly to immobilize RNA. RNA was crosslinked to the membrane by exposure to 254 nm ultraviolet light for 1–2 min. The membrane was blocked with 5% skim milk in TBST buffer at room temperature for 1 h, followed by overnight incubation with a specific anti‐m^6^A monoclonal antibody at 4°C. After washes with TBST buffer, the membrane was incubated with an HRP‐conjugated secondary antibody (1:10,000 dilution) at room temperature for 1 h. The membrane was washed three times with PBST buffer, and the chemiluminescent substrate was applied. Signals were captured using a chemiluminescence imaging system (Bio‐Rad ChemiDoc), and m^6^A levels were quantified based on signal intensity. After fluorescence imaging, the membrane was stained with 0.2% methylene blue staining buffer for 30 min with gentle shaking. Wash the membrane with dH_2_O, and image with an imaging system (Bio‐Rad ChemiDoc).

### Assessment of mRNA Decay

BMDMs cultured in 24‐well plates were added fresh medium that contained actinomycin D at a final concentration of 5 ug ml^−1^. Total cellular RNAs were extracted at 0, 2, 4, 6, and 8 hs after actinomycin D treatment, and mRNA transcripts at each time point were quantified by RT‐qPCR. The mRNA level at each time point was normalized to that at 0 h, and the changes were plotted against time.

### Luciferase assay

The region surrounding the identified m^6^A site in the 3’‐UTR of Slc15a3 mRNA (wild‐type sequence or A‐to‐C mutation) was cloned into the pmirGLO Dual‐Luciferase expression vector (Promega). Vector (500 ng) was transfected in Raw264.7 and THP‐1 cells in a 6‐well plate with Lipofectamine 3000, and cells were lysed after 48 h. Firefly luciferase signals were measured with a luminometer and normalized to Renilla luciferase activity with a Dual‐Glo Luciferase Assay system.

### Human Subjects

Collect fresh peripheral blood from healthies and patients into vacuum tubes containing anticoagulants (heparin/EDTA). Dilute the blood sample 1:1 with phosphate‐buffered saline (PBS). Layer 4 mL of diluted blood carefully over 4 mL Ficoll‐Paque PREMIUM density gradient medium in a 15 mL conical tube. Centrifuge at 400 × g for 25 min at room temperature with brake disabled. Aspirate the buffy coat layer containing PBMCs using a sterile transfer pipette. Wash cells twice with PBS (10 mL) by centrifugation at 300 ×g for 10 min Resuspend the cell pellet in complete culture medium. To detect M1 polarization, cells were stained with CD86 and ROS, which were analyzed on a BD LSRFortessa flow cytometer.

Punch biopsies of psoriatic skin were obtained from patients under local lidocaine anesthesia. Normal skin specimens were acquired from healthy donors undergoing plastic surgery. All participants were thoroughly informed about the study's objectives, procedures, and potential risks, and written informed consent in Chinese was obtained prior to inclusion. The study was approved by the Ethics Committee of the Dermatology Hospital of Southern Medical University.

### Statistical Analysis

Statistical analysis was performed using GraphPad Prism 8. Unpaired t‐test, one‐phase decay, and correlation analyses were employed. For two‐group comparisons unpaired two‐tailed Student's *t*‐test was used, and for three or more group comparisons, ordinary one‐way or two‐way analysis of variance (ANOVA) was performed. Data were presented as mean ± S.E.M. or S.D., with *P* ≤ 0.05 considered statistically significant.

### Ethics Approval and Consent to Participate

This study was approved by the Ethics Committee of the Dermatology Hospital of Southern Medical University. The objectives, procedures, and potential risks were verbally explained to all participants. Written informed consent (in Chinese) was obtained from all participants before inclusion in the study.

## Conflict of Interest

The authors declare no conflict of interest.

## Author Contributions

T.H. S.C., and K.D. contributed equally to this work. Z.R., Y.L., and T.H. conceived the study, designed the experiments, analyzed the data, and wrote the manuscript. T.H. and S.C. performed most of the experiments. X.Z. constructed the plasmids and performed the RNAi. K.D. performed the staining assay. L.Y. collected human samples. D.M. performed MeRIP‐seq. K.C. and Y.L. performed the bioinformatics analysis for MeRIP‐seq. W.L. and K.D. performed mouse breeding and genotyping. X.W. performed some data analysis. G.L. supervised the MeRIP‐seq experiments and bioinformatics analysis. Z.R., Y.L., and B.Y. supervised the study.

## Supporting information



Supporting Information

Supporting Information

Supporting Information

## Data Availability

The MeRIP‐Seq data have been deposited in the NCBI BioProject database under the accession number GSE274791.
