# Supplementary material for: METTL3/ALKBH5‐Mediated N6‐Methyladenosine Modification Drives Macrophage M1 Polarization via the SLC15A3‐TASL‐IRF5 Signaling Axis in Psoriasis
Source: Adv Sci (Weinh). 2025 Jul 18;12(36):e01408. doi: 10.1002/advs.202501408 (PMC12462922; doi:10.1002/advs.202501408)
Supplement: Supplementary file 1 — Supporting Information [file ADVS-12-e01408-s001.pot]

## Slide 1
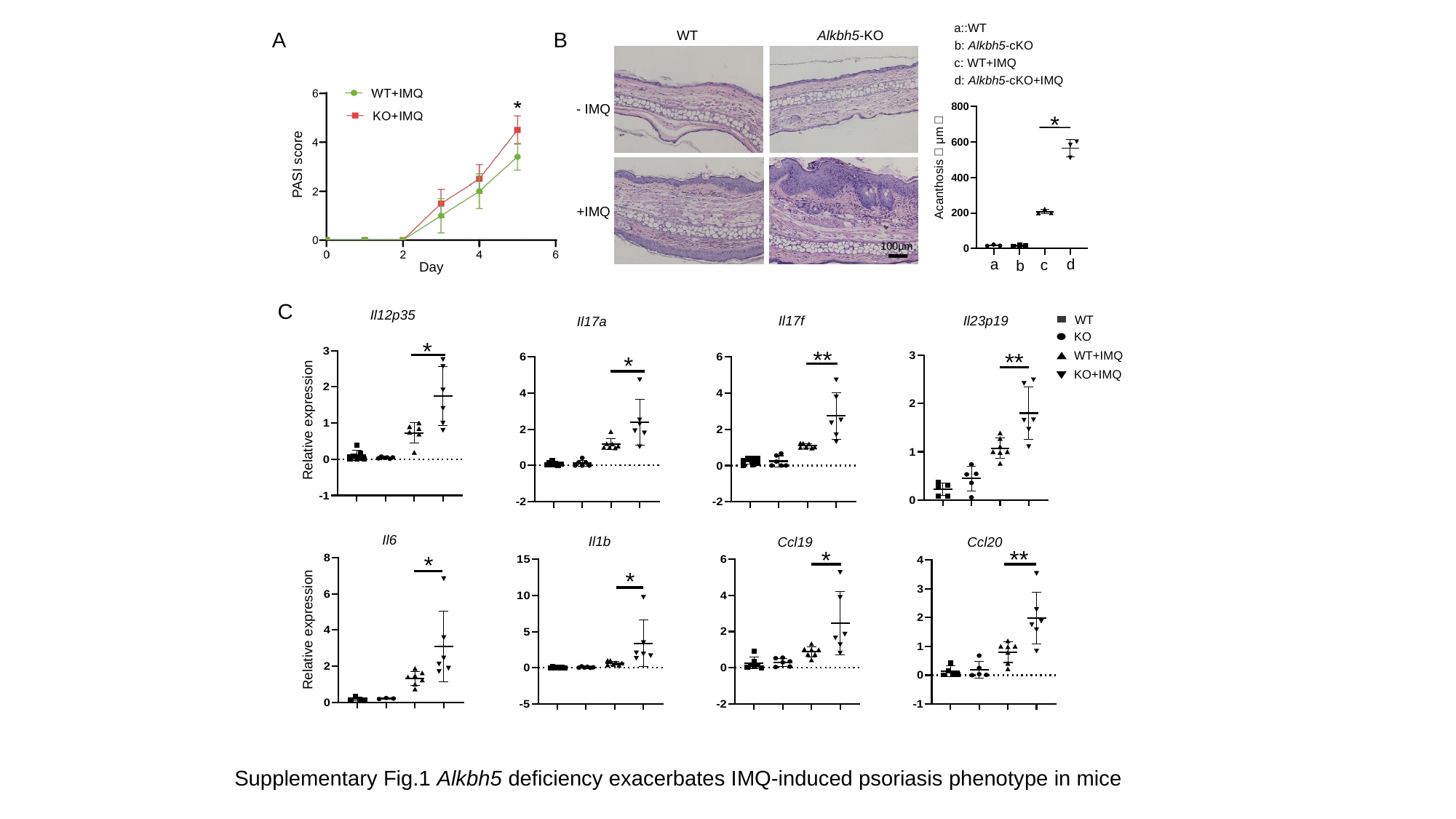

a::WT
b: Alkbh5-cKO
c: WT+IMQ
d: Alkbh5-cKO+IMQ
A
PASI score
Day
B
WT
Alkbh5-KO
- IMQ
+IMQ
100μm
*
Acanthosis（μm）
a
d
c
b
C
Il12p35
*
Relative expression
Il17f
**
Il23p19
**
Il17a
*
WT
KO
WT+IMQ
KO+IMQ
Il6
*
Relative expression
Il1b
*
Ccl19
*
Ccl20
**
Supplementary Fig.1 Alkbh5 deficiency exacerbates IMQ-induced psoriasis phenotype in mice

## Slide 2
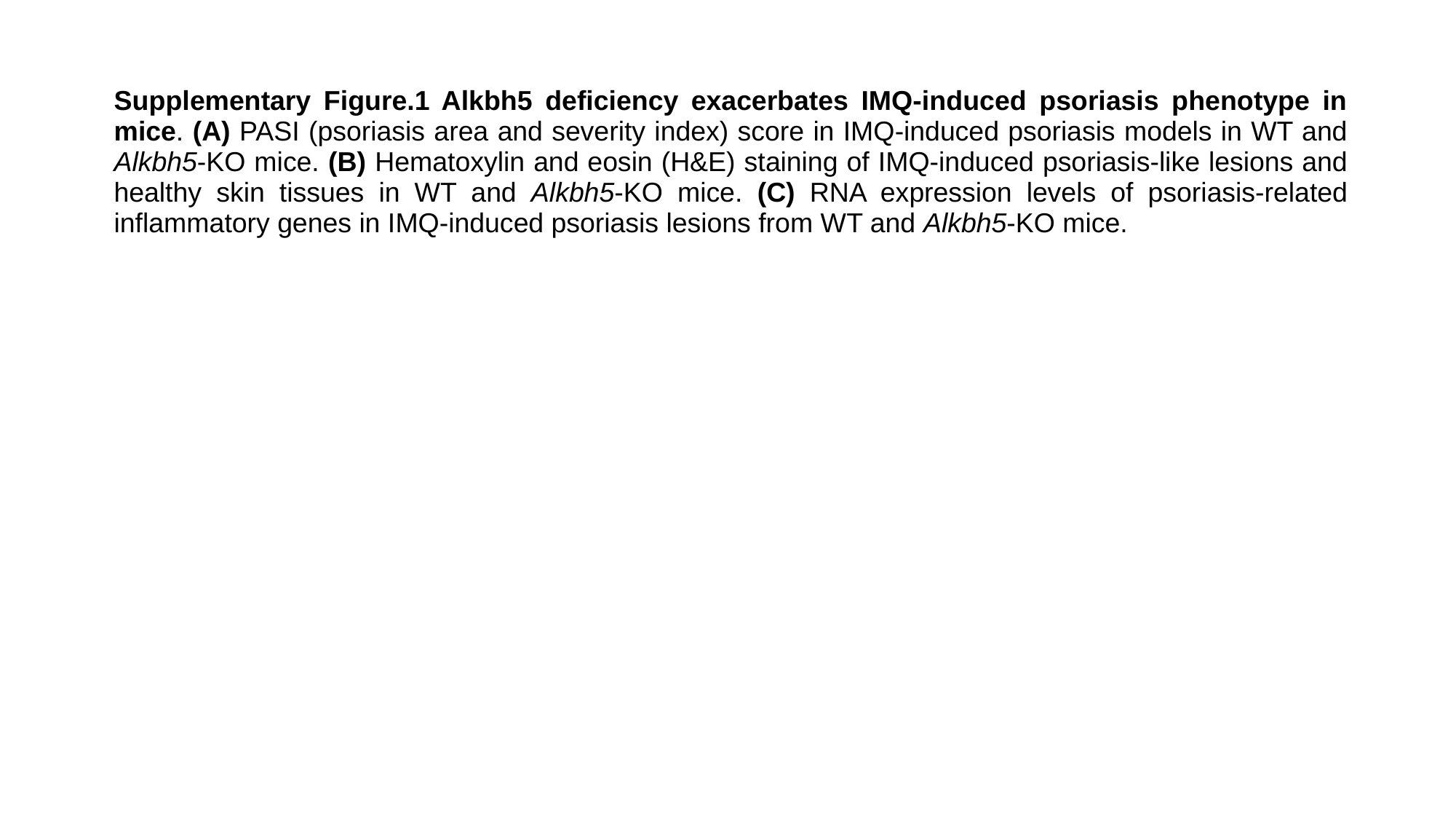

Supplementary Figure.1 Alkbh5 deficiency exacerbates IMQ-induced psoriasis phenotype in mice. (A) PASI (psoriasis area and severity index) score in IMQ-induced psoriasis models in WT and Alkbh5-KO mice. (B) Hematoxylin and eosin (H&E) staining of IMQ-induced psoriasis-like lesions and healthy skin tissues in WT and Alkbh5-KO mice. (C) RNA expression levels of psoriasis-related inflammatory genes in IMQ-induced psoriasis lesions from WT and Alkbh5-KO mice.

## Slide 3
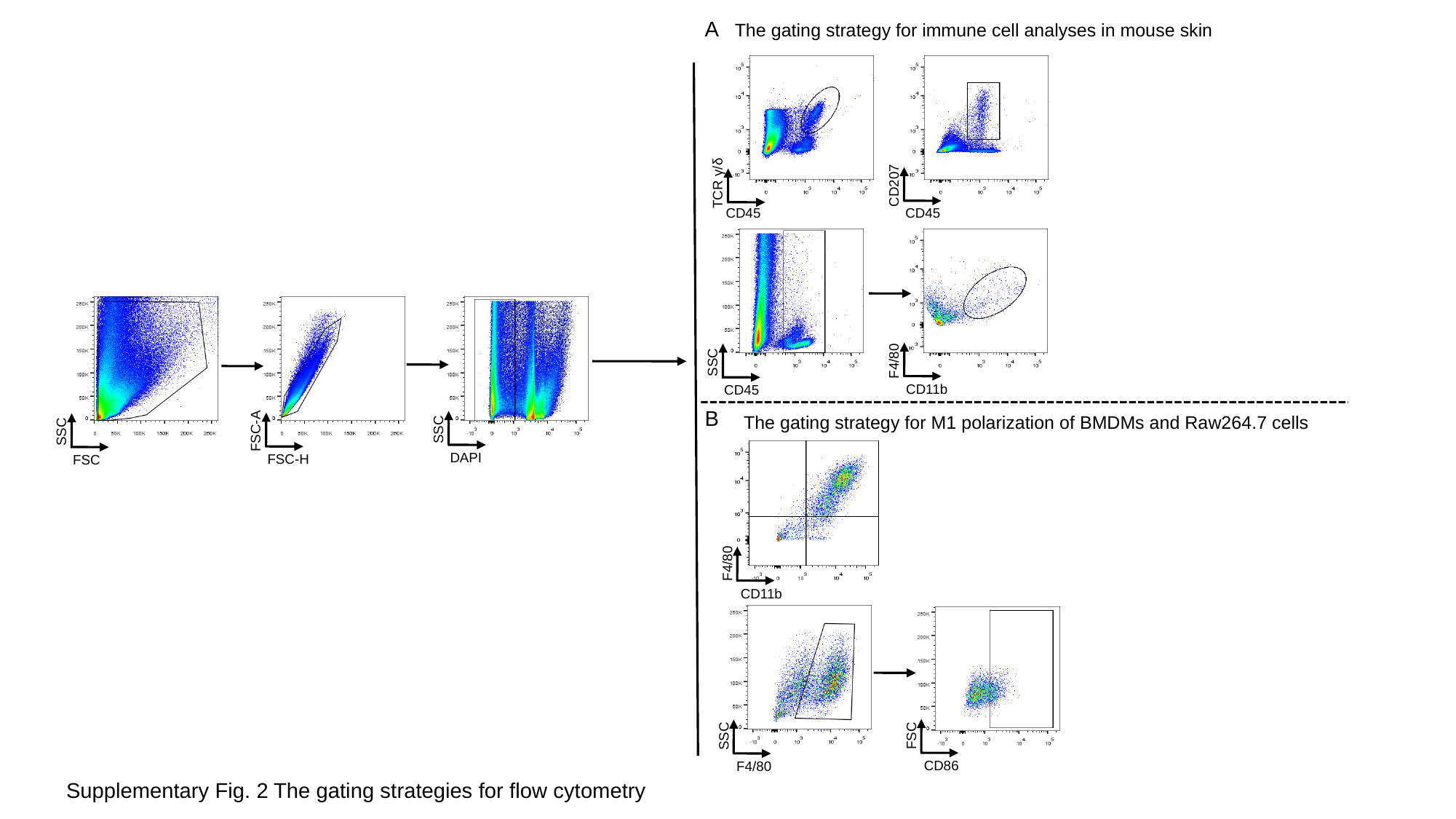

A
The gating strategy for immune cell analyses in mouse skin
CD207
CD45
TCR γ/δ
CD45
SSC
CD45
F4/80
CD11b
SSC
FSC
FSC-A
FSC-H
SSC
DAPI
B
The gating strategy for M1 polarization of BMDMs and Raw264.7 cells
F4/80
CD11b
SSC
F4/80
FSC
CD86
Supplementary Fig. 2 The gating strategies for flow cytometry

## Slide 4
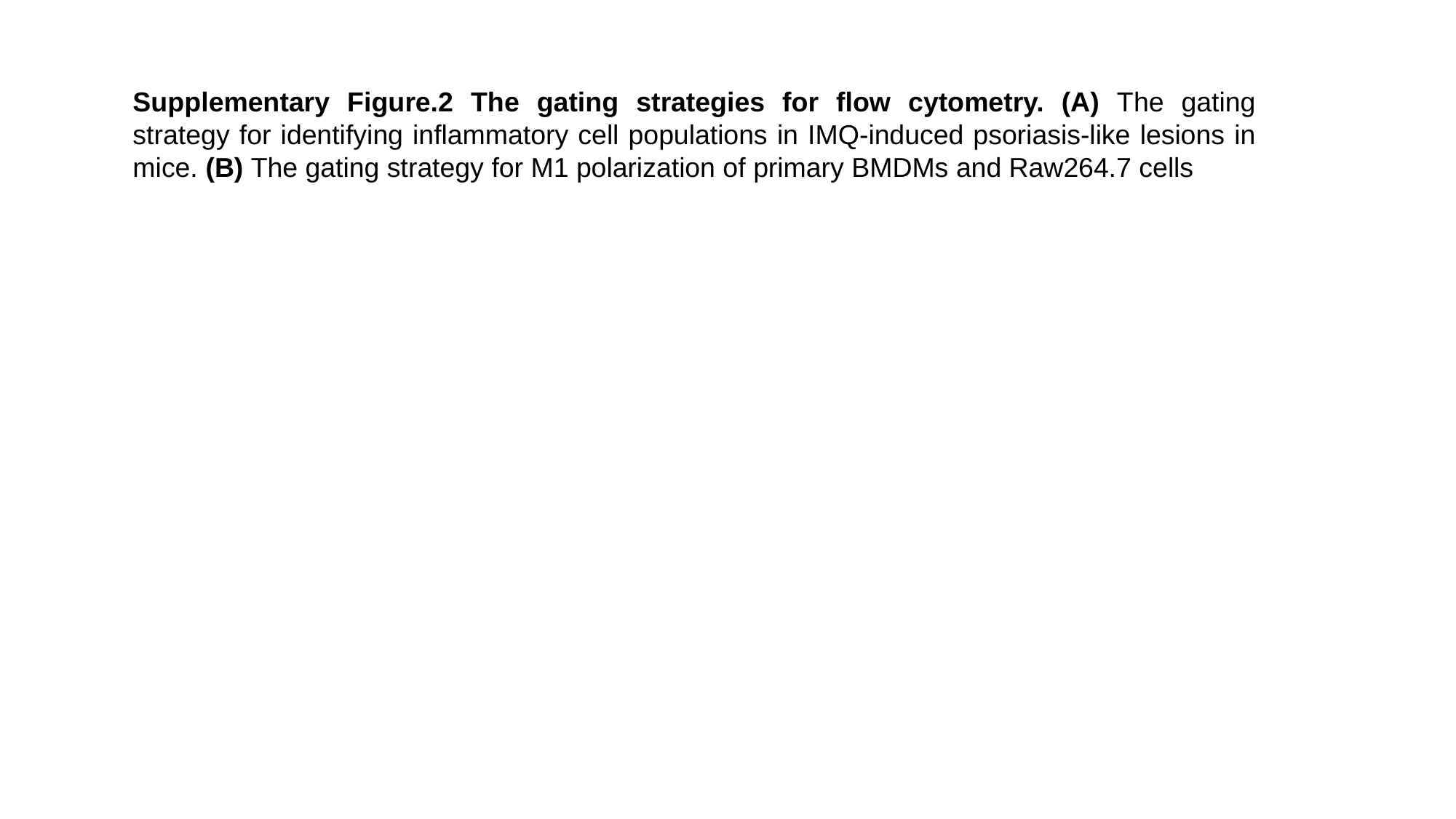

Supplementary Figure.2 The gating strategies for flow cytometry. (A) The gating strategy for identifying inflammatory cell populations in IMQ-induced psoriasis-like lesions in mice. (B) The gating strategy for M1 polarization of primary BMDMs and Raw264.7 cells

## Slide 5
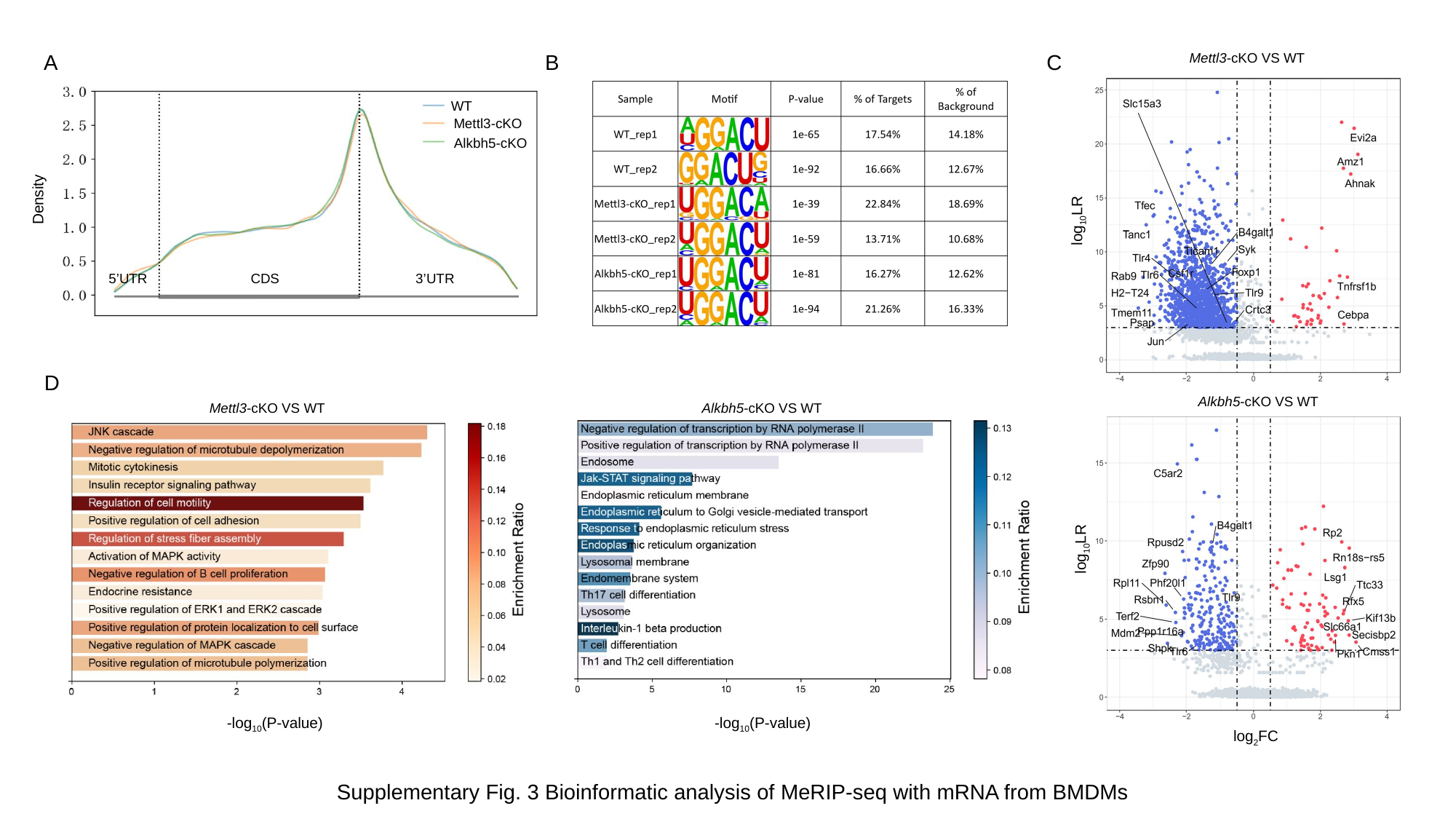

A
B
C
Mettl3-cKO VS WT
WT
Mettl3-cKO
Alkbh5-cKO
Density
5’UTR
CDS
3’UTR
log10LR
D
Alkbh5-cKO VS WT
Mettl3-cKO VS WT
Alkbh5-cKO VS WT
log10LR
-log10(P-value)
-log10(P-value)
log2FC
Supplementary Fig. 3 Bioinformatic analysis of MeRIP-seq with mRNA from BMDMs

## Slide 6
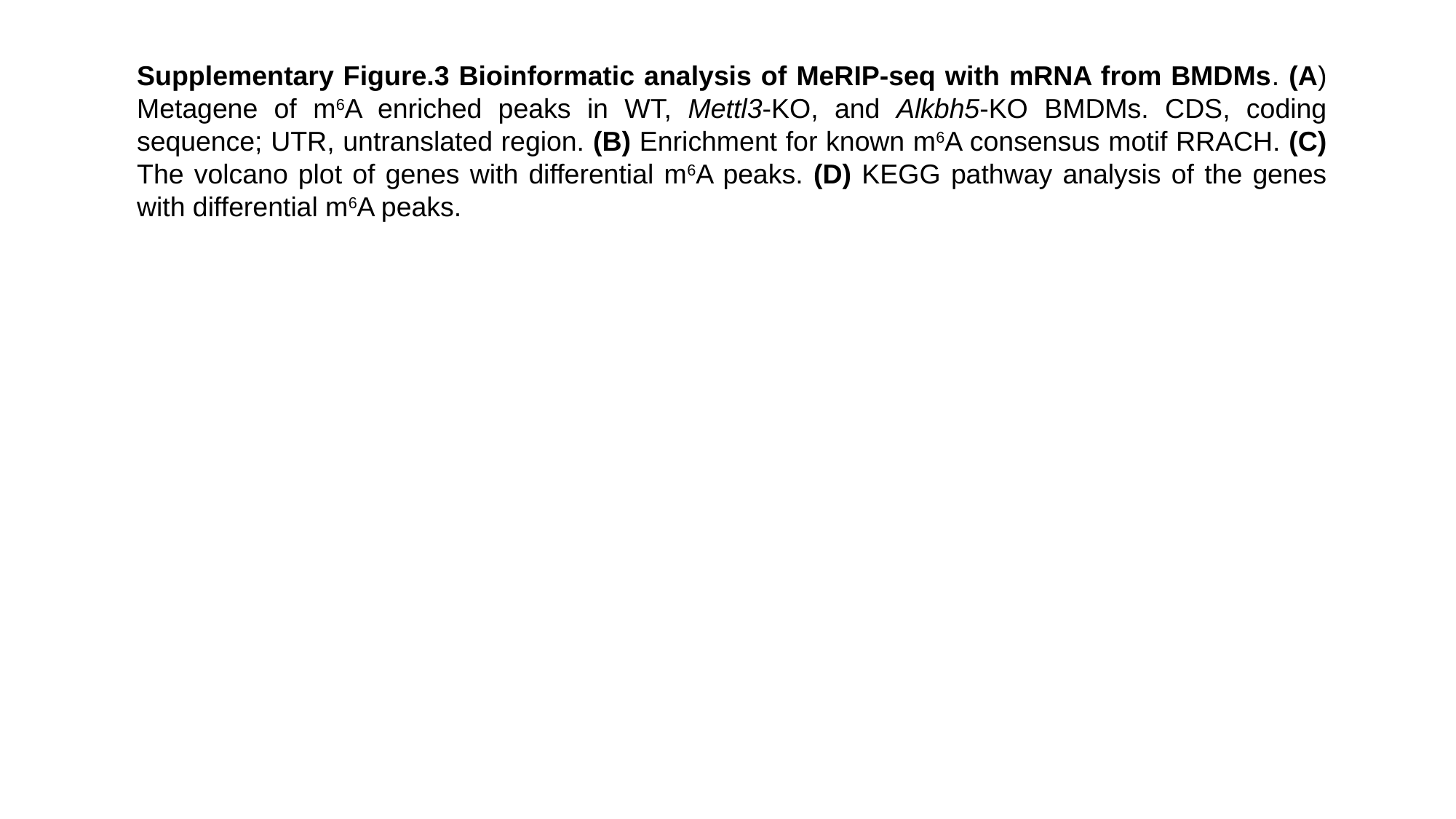

Supplementary Figure.3 Bioinformatic analysis of MeRIP-seq with mRNA from BMDMs. (A) Metagene of m6A enriched peaks in WT, Mettl3-KO, and Alkbh5-KO BMDMs. CDS, coding sequence; UTR, untranslated region. (B) Enrichment for known m6A consensus motif RRACH. (C) The volcano plot of genes with differential m6A peaks. (D) KEGG pathway analysis of the genes with differential m6A peaks.

## Slide 7
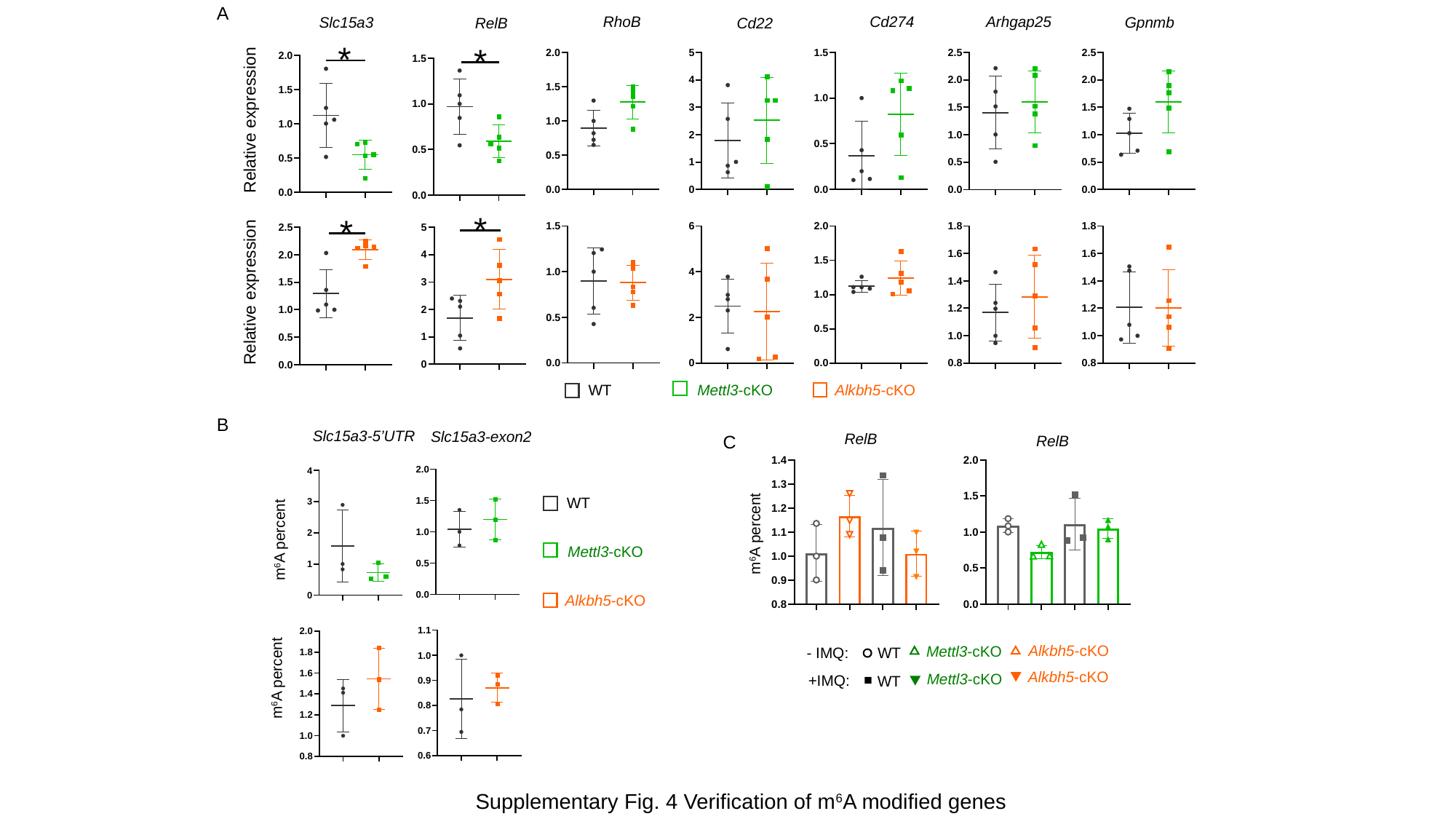

A
RhoB
Cd274
Arhgap25
Gpnmb
Slc15a3
Cd22
RelB
Relative expression
Relative expression
WT
Mettl3-cKO
Alkbh5-cKO
B
Slc15a3-5’UTR
Slc15a3-exon2
m6A percent
m6A percent
RelB
C
RelB
m6A percent
WT
Mettl3-cKO
Alkbh5-cKO
Alkbh5-cKO
Mettl3-cKO
- IMQ:
WT
Alkbh5-cKO
Mettl3-cKO
+IMQ:
WT
Supplementary Fig. 4 Verification of m6A modified genes

## Slide 8
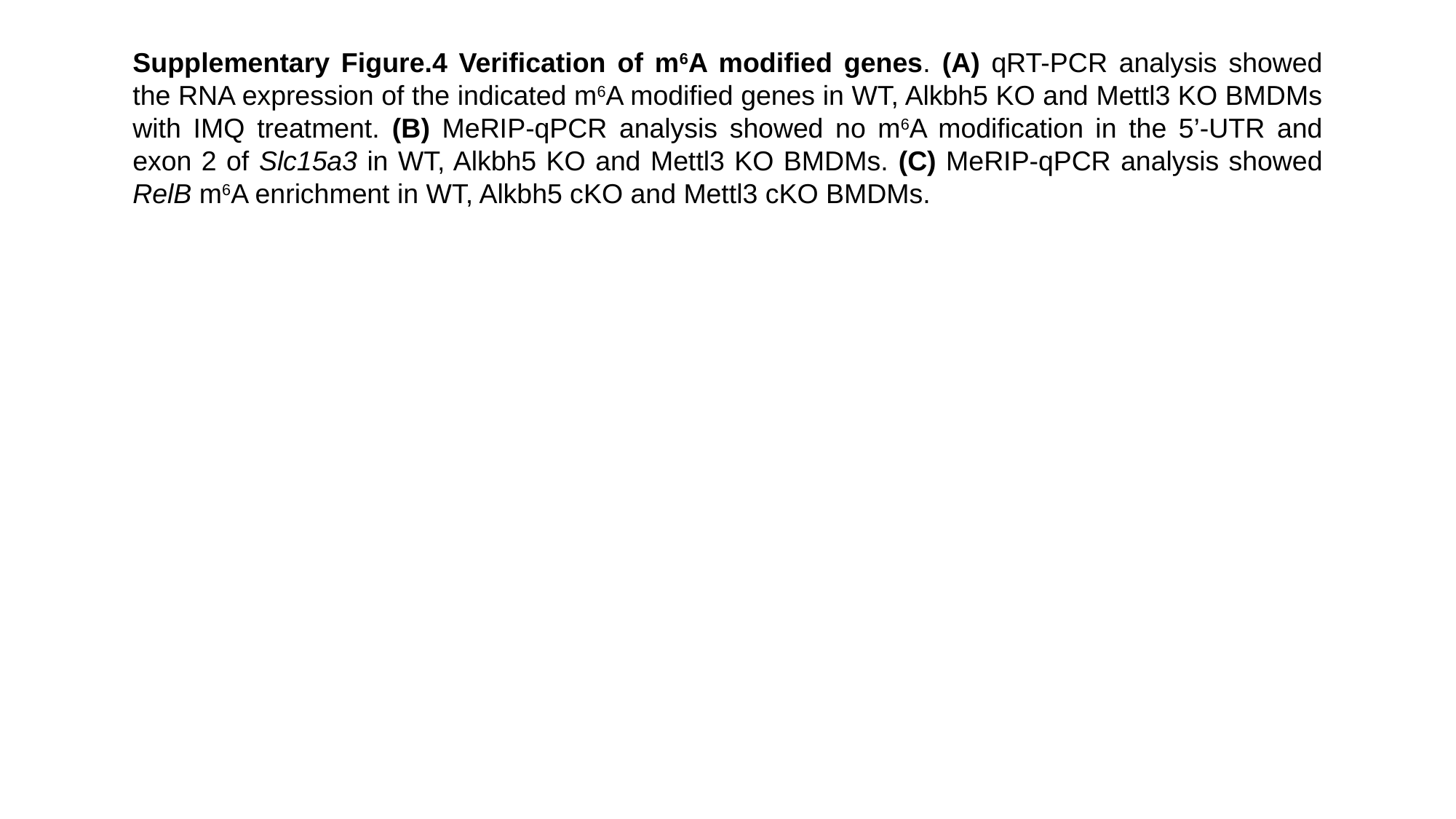

Supplementary Figure.4 Verification of m6A modified genes. (A) qRT-PCR analysis showed the RNA expression of the indicated m6A modified genes in WT, Alkbh5 KO and Mettl3 KO BMDMs with IMQ treatment. (B) MeRIP-qPCR analysis showed no m6A modification in the 5’-UTR and exon 2 of Slc15a3 in WT, Alkbh5 KO and Mettl3 KO BMDMs. (C) MeRIP-qPCR analysis showed RelB m6A enrichment in WT, Alkbh5 cKO and Mettl3 cKO BMDMs.

## Slide 9
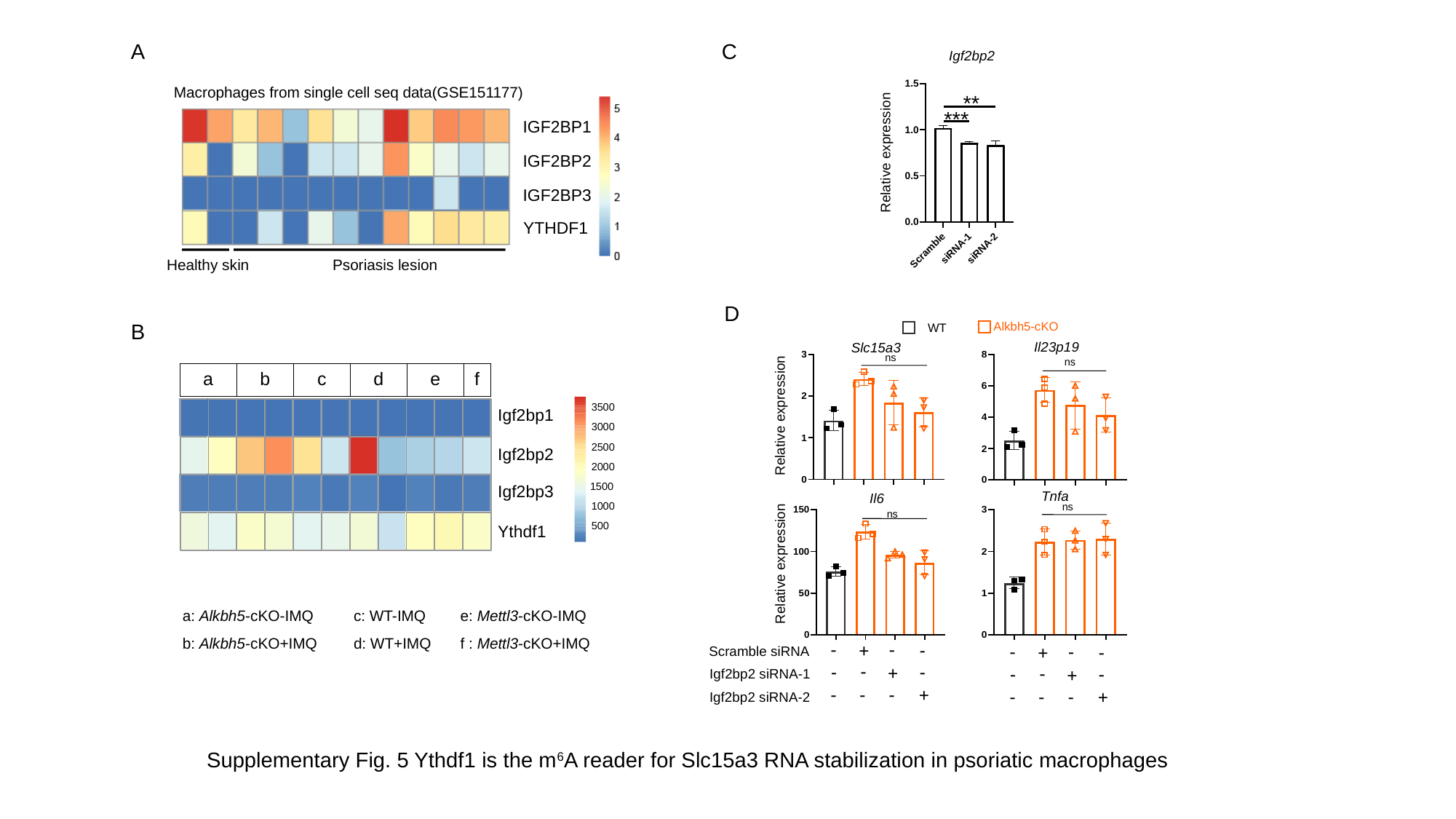

A
C
Igf2bp2
**
***
Macrophages from single cell seq data(GSE151177)
IGF2BP1
IGF2BP2
IGF2BP3
YTHDF1
Psoriasis lesion
Healthy skin
Relative expression
D
B
Alkbh5-cKO
WT
Il23p19
Slc15a3
ns
ns
| a | b | c | d | e |
| --- | --- | --- | --- | --- |
| f |
| --- |
3500
3000
2500
2000
1500
1000
500
Igf2bp1
Relative expression
Igf2bp2
Igf2bp3
Tnfa
Il6
ns
ns
Ythdf1
Relative expression
a: Alkbh5-cKO-IMQ
b: Alkbh5-cKO+IMQ
c: WT-IMQ
d: WT+IMQ
e: Mettl3-cKO-IMQ
f : Mettl3-cKO+IMQ
-
-
-
-
+
-
-
-
+
+
-
-
-
-
-
-
+
-
-
-
+
+
-
-
Scramble siRNA
Igf2bp2 siRNA-1
Igf2bp2 siRNA-2
Supplementary Fig. 5 Ythdf1 is the m6A reader for Slc15a3 RNA stabilization in psoriatic macrophages

## Slide 10
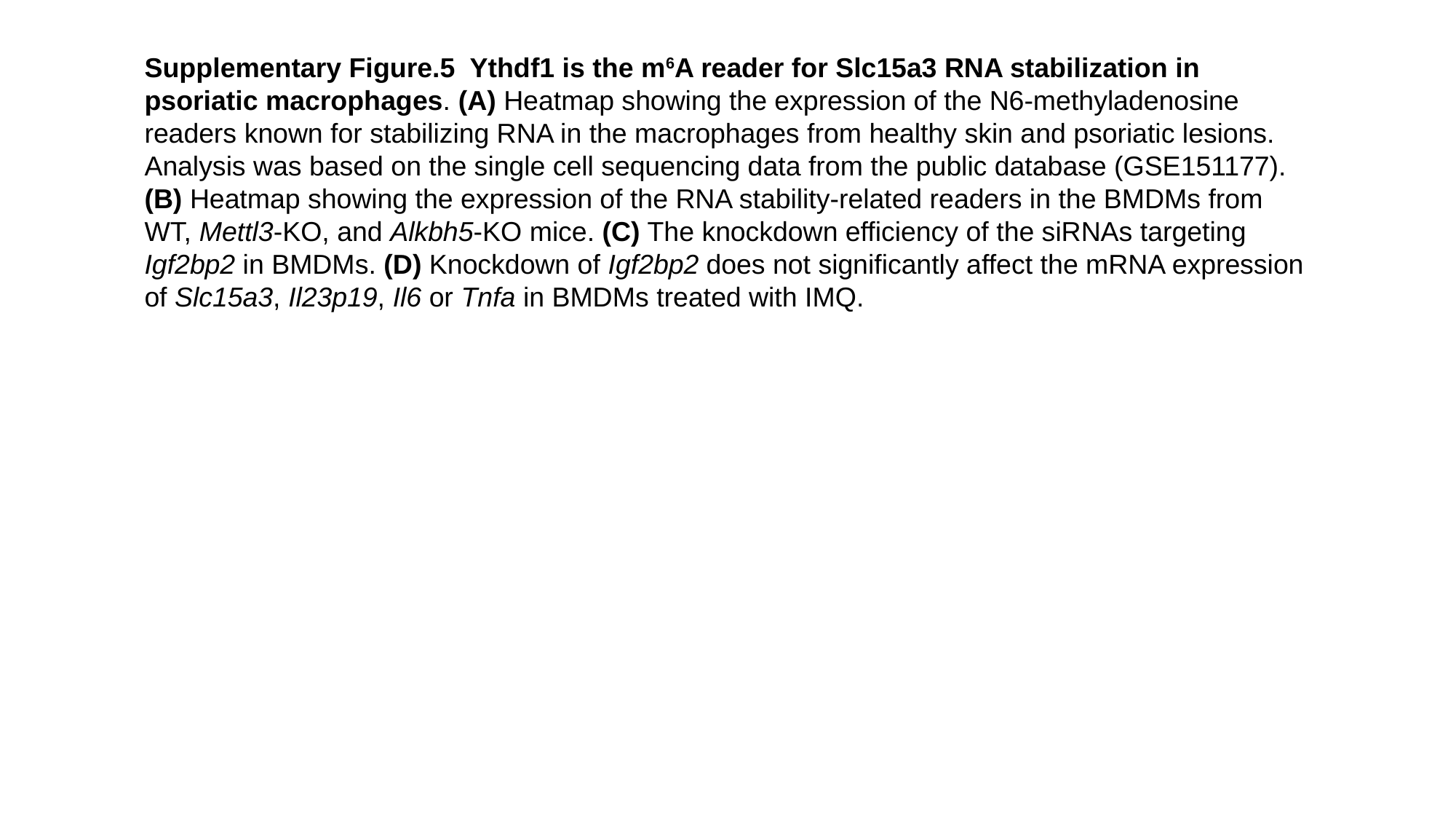

Supplementary Figure.5 Ythdf1 is the m6A reader for Slc15a3 RNA stabilization in psoriatic macrophages. (A) Heatmap showing the expression of the N6-methyladenosine readers known for stabilizing RNA in the macrophages from healthy skin and psoriatic lesions. Analysis was based on the single cell sequencing data from the public database (GSE151177). (B) Heatmap showing the expression of the RNA stability-related readers in the BMDMs from WT, Mettl3-KO, and Alkbh5-KO mice. (C) The knockdown efficiency of the siRNAs targeting Igf2bp2 in BMDMs. (D) Knockdown of Igf2bp2 does not significantly affect the mRNA expression of Slc15a3, Il23p19, Il6 or Tnfa in BMDMs treated with IMQ.

## Slide 11
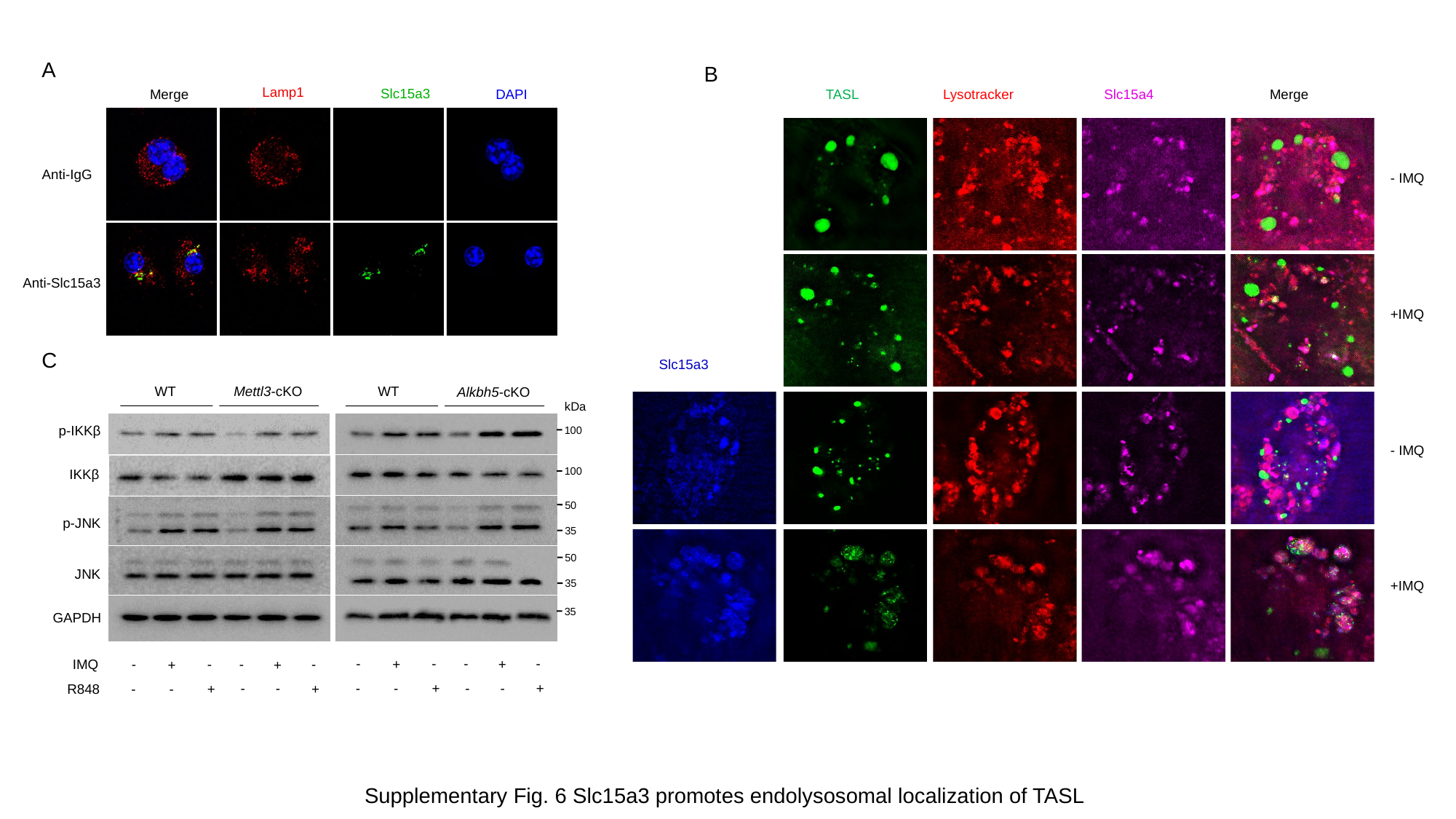

A
B
Lamp1
Slc15a3
Merge
DAPI
TASL
Lysotracker
Slc15a4
Merge
- IMQ
+IMQ
Slc15a3
- IMQ
+IMQ
Anti-IgG
Anti-Slc15a3
C
WT
Mettl3-cKO
WT
Alkbh5-cKO
p-IKKβ
IKKβ
p-JNK
JNK
GAPDH
-
-
-
-
+
+
-
-
-
-
+
+
-
-
-
-
+
+
-
-
-
-
+
+
IMQ
R848
100
100
50
35
50
35
35
kDa
Supplementary Fig. 6 Slc15a3 promotes endolysosomal localization of TASL

## Slide 12
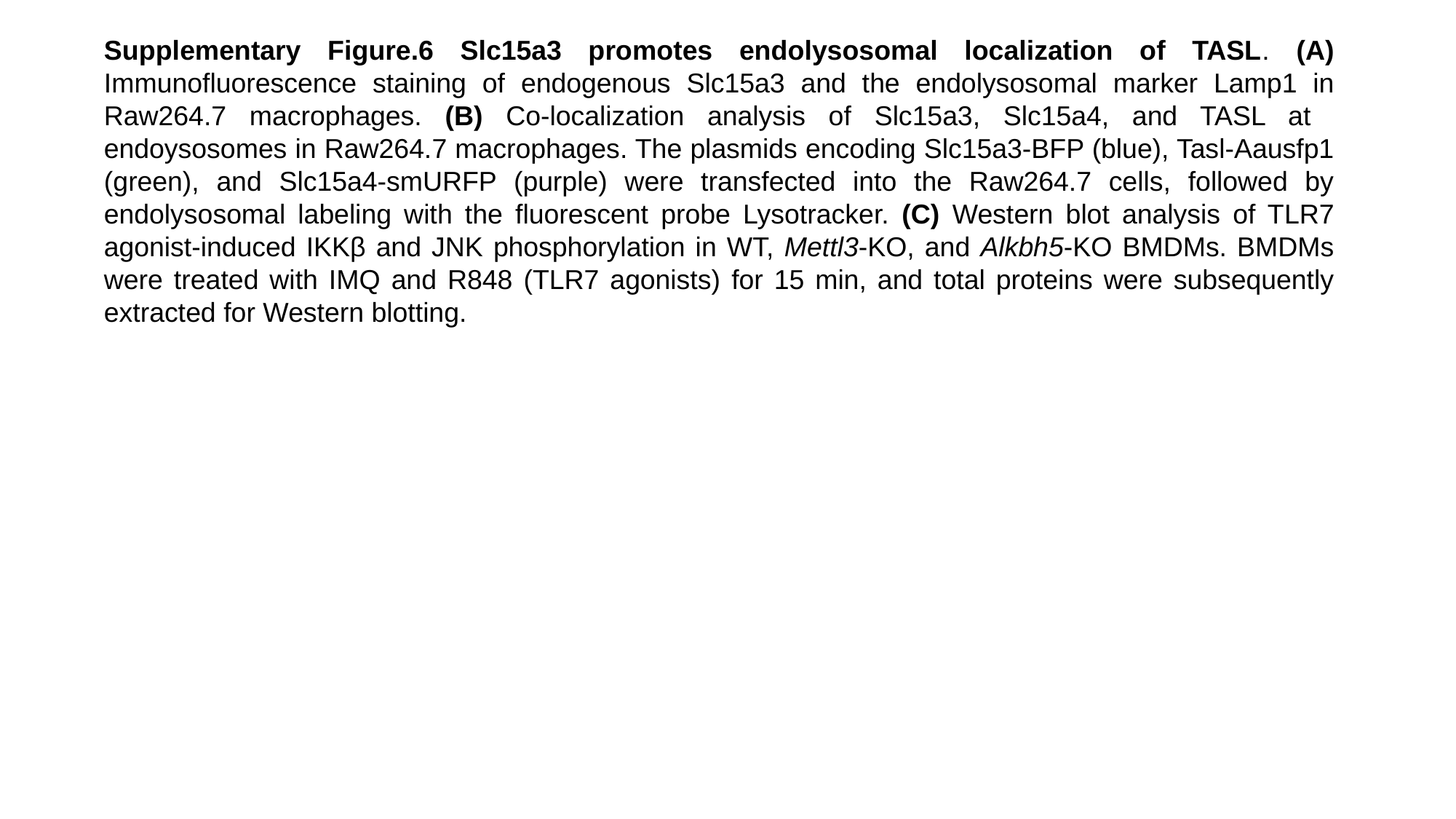

Supplementary Figure.6 Slc15a3 promotes endolysosomal localization of TASL. (A) Immunofluorescence staining of endogenous Slc15a3 and the endolysosomal marker Lamp1 in Raw264.7 macrophages. (B) Co-localization analysis of Slc15a3, Slc15a4, and TASL at endoysosomes in Raw264.7 macrophages. The plasmids encoding Slc15a3-BFP (blue), Tasl-Aausfp1 (green), and Slc15a4-smURFP (purple) were transfected into the Raw264.7 cells, followed by endolysosomal labeling with the fluorescent probe Lysotracker. (C) Western blot analysis of TLR7 agonist-induced IKKβ and JNK phosphorylation in WT, Mettl3-KO, and Alkbh5-KO BMDMs. BMDMs were treated with IMQ and R848 (TLR7 agonists) for 15 min, and total proteins were subsequently extracted for Western blotting.

## Slide 13
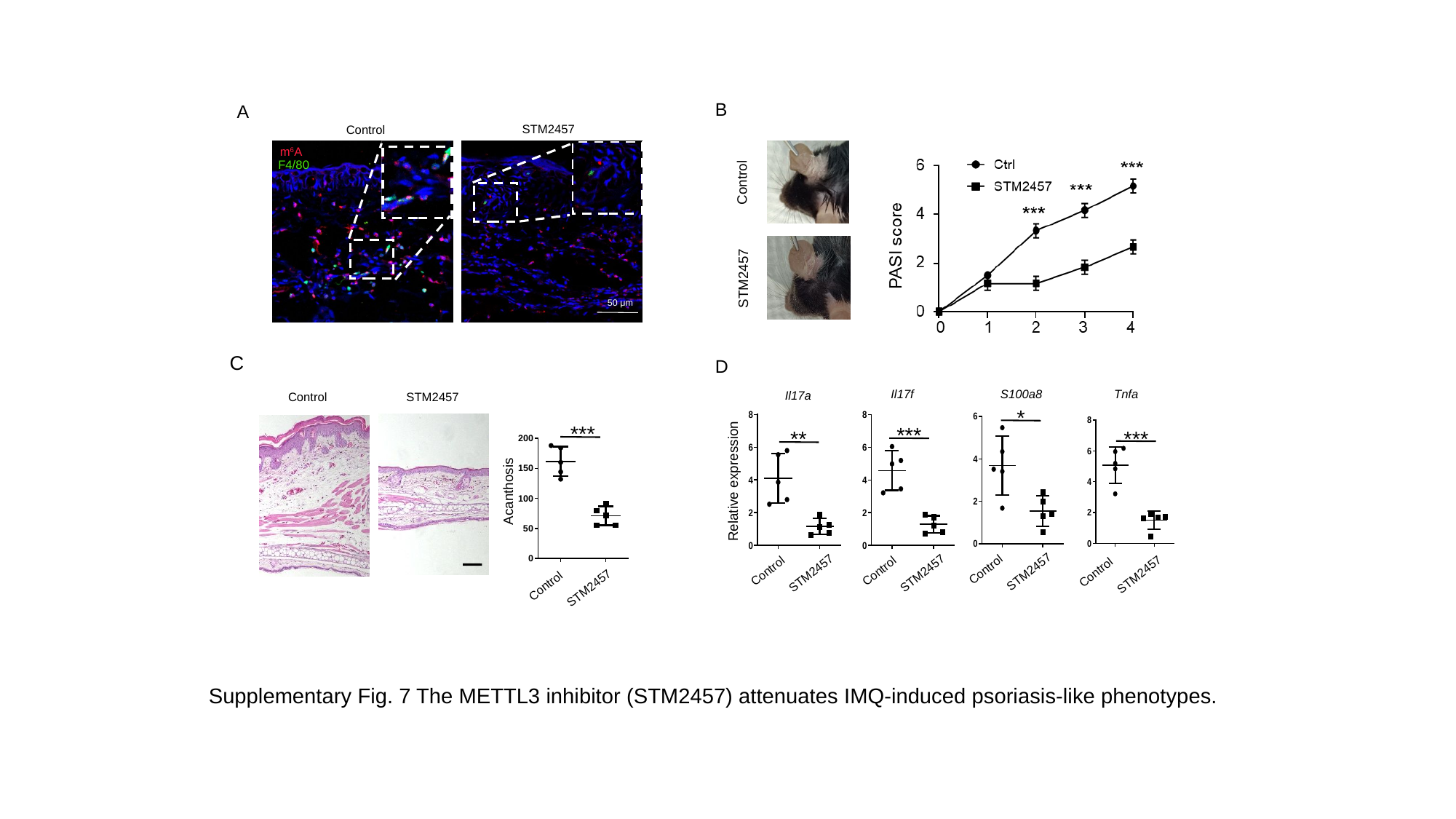

B
A
STM2457
Control
50 μm
m6A
F4/80
Control
STM2457
C
D
Il17f
S100a8
Tnfa
Il17a
 *
 ***
 **
 ***
Relative expression
Control
STM2457
 ***
Acanthosis
Control
STM2457
Control
STM2457
Control
STM2457
Control
STM2457
Control
STM2457
Supplementary Fig. 7 The METTL3 inhibitor (STM2457) attenuates IMQ-induced psoriasis-like phenotypes.

## Slide 14
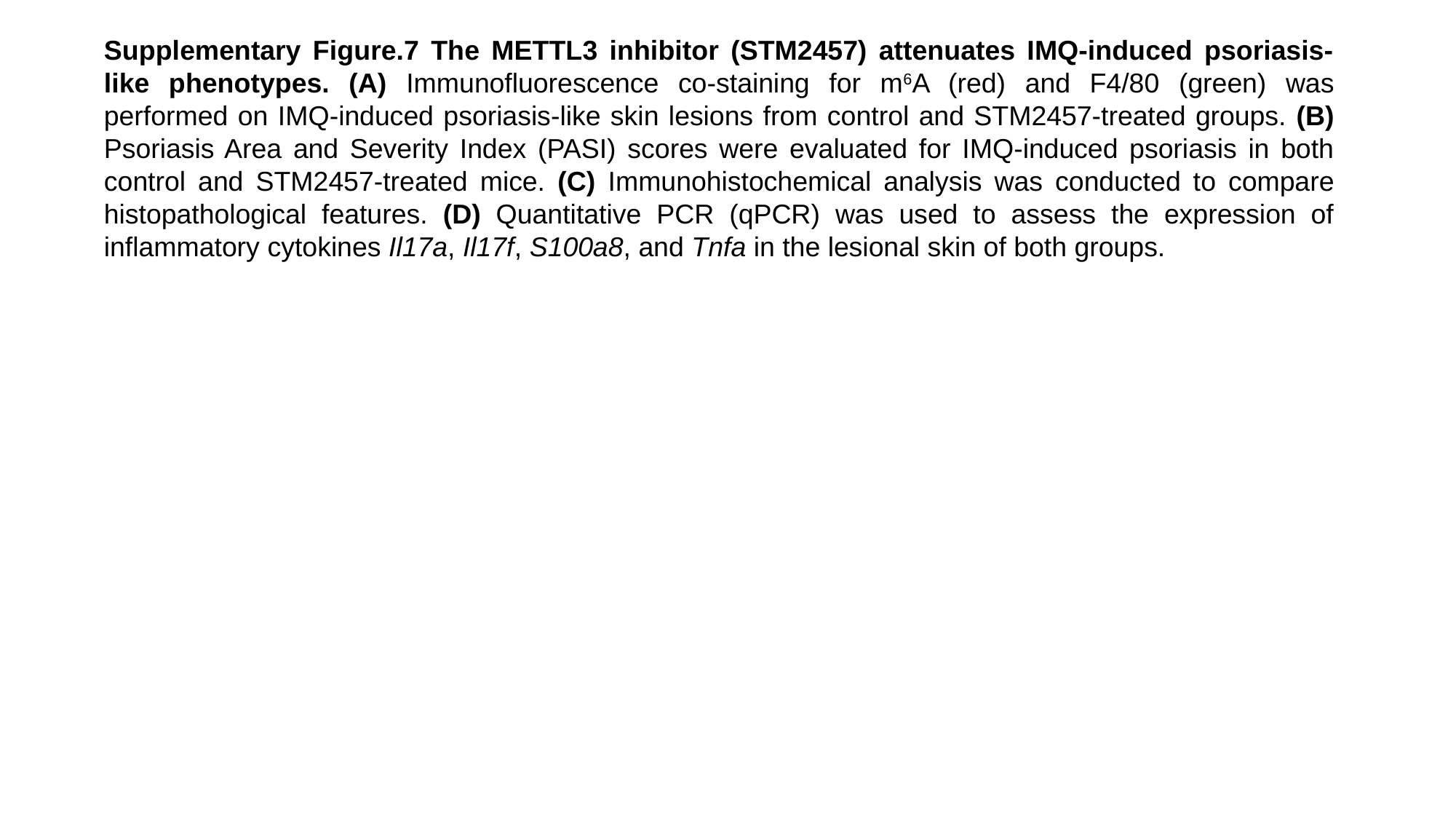

Supplementary Figure.7 The METTL3 inhibitor (STM2457) attenuates IMQ-induced psoriasis-like phenotypes. (A) Immunofluorescence co-staining for m6A (red) and F4/80 (green) was performed on IMQ-induced psoriasis-like skin lesions from control and STM2457-treated groups. (B) Psoriasis Area and Severity Index (PASI) scores were evaluated for IMQ-induced psoriasis in both control and STM2457-treated mice. (C) Immunohistochemical analysis was conducted to compare histopathological features. (D) Quantitative PCR (qPCR) was used to assess the expression of inflammatory cytokines Il17a, Il17f, S100a8, and Tnfa in the lesional skin of both groups.
