# Supplementary material for: METTL3/ALKBH5‐Mediated N6‐Methyladenosine Modification Drives Macrophage M1 Polarization via the SLC15A3‐TASL‐IRF5 Signaling Axis in Psoriasis
Source: Adv Sci (Weinh). 2025 Jul 18;12(36):e01408. doi: 10.1002/advs.202501408 (PMC12462922; doi:10.1002/advs.202501408)
Supplement: Supplementary file 3 — Supporting Information [file ADVS-12-e01408-s003.docx]

**Supplementary Table 2: Plasmid information**

1. **pCAG-TASL-MYC-2A-mCherry-pA**

atgctatcagaaggatatctcagtggacttacctactggaatgacattcattggaattgtgcatcttataatgaaccggtggctggggaccaaggcaaagagacaagttctgttgctgctctttcatattcctctgtggatgaaacacaagttcaaagtctttatgtgagctgcaaatcctctgggaagtttatttcatcagtgcatgcaagggcgagtcagcacagcagaagccagagcagaacagtgctgcaggcaaacagcaaccctgtatttgaaagtccaactttagctgcagttggtatatgcagagatgtgatcagggagacctacttggttccaccttcttgtaaaagtatttgcaaaaattacaacgacttacatattgcagggggacaggtgatggccattaactcagtaatggcaaatttcccctctgagagcagctttgaagatggtcctttgctaaagtcatctgagatttctttgtccatggaggattccacttccactcagctcactgaacttcccctcaaacctatccagcggtactcatcctactggaggataaccagcatcaaagagaaaagcagcctgcaaatgcagaagcctatttcaaatgcagtgctcaatgagtacctggagcagaaggtggtggaattgtataagcaatatattatggacactgtgtttcatgacagttctcctacccagattctggcatcagaattcatcatgacgaatgtagatcaaattagtcttcaagtgtctaaagagaagaacctggacacttcaaaagtcaaggacatagttattagccacctgttgcagttggtatcatctgagatcagcacccctagtcttcatatttctcagtatagcaatataactccaaccggtggatccgaacaaaaactcatctcagaagaggatctgctcgagagcaccggtggcagcggagctactaacttcagcctgctgaagcaggctggagacgtggaggagaaccctggacctgccggtatggtgagcaagggcgaggaggataacatggccatcatcaaggagttcatgcgcttcaaggtgcacatggagggctccgtgaacggccacgagttcgagatcgagggcgagggcgagggccgcccctacgagggcacccagaccgccaagctgaaggtgaccaagggtggccccctgcccttcgcctgggacatcctgtcccctcagttcatgtacggctccaaggcctacgtgaagcaccccgccgacatccccgactacttgaagctgtccttccccgagggcttcaagtgggagcgcgtgatgaacttcgaggacggcggcgtggtgaccgtgacccaggactcctccctgcaggacggcgagttcatctacaaggtgaagctgcgcggcaccaacttcccctccgacggccccgtaatgcagaagaagaccatgggctgggaggcctcctccgagcggatgtaccccgaggacggcgccctgaagggcgagatcaagcagaggctgaagctgaaggacggcggccactacgacgctgaggtcaagaccacctacaaggccaagaagcccgtgcagctgcccggcgcctacaacgtcaacatcaagttggacatcacctcccacaacgaggactacaccatcgtggaacagtacgaacgcgccgagggccgccactccaccggcggcatggacgagctgtacaagtaa

1. **pCAG-Slc15a3-HA-2A-mCherry-pA**

atggggtctgcccctcgcgcggaagagcaaccaagcaggtccggagagcgacaaccgctggtggcccgaggcccgcggggaccacgacggtggagacggacagctgctgctgcggtgctgctagtgcagatgctggaacgcgctgccttctttggtgtcacctccaacctcgtgctctacctcaacagcttgaacttcaattgggacggccaacaggcatcacgggccacactcctcttcctaggcgcctcctacctgctggctccagtggggggctggctggcagacgtgtacctgggacgcttcctcactatctcgctcagcctgctgctctatctggctgcctctggcttgttgctcaccaccatcaccaatgatggccgcagatccttctgtggagagatgcccgagttgccactggaacctgcctgcccatcctcaagctgccaaggctcctggtccagcccctactgcgcgaccaccctctacttggtgttgctgctcctggccctggctgccagctccgtcaggagcaccctcacctcgttcggggctgaccaggtgatggatctcgggcgcgatgccacccgccgcttcttcaactggttctattggagcatcaacctgggtgccatattgtccctgttggtggtggctttcatcgagcagaacatcagcttcctatggggctacagcatcatcgtgggcctcgtgggcctggcattcttcatttttctctttgccacaccagtcttcatcaccaagcccccaacaggcagccaagtgtcatctatgctgaagcttgcgttccaaaactgctgtccctgccggaggtcctcttccagggactctgaaagtgcccacctgttgcctgaccagaggtctaaccagcctggaccttctccacaagaagatatggccaacttccaggtgctggtgaagatcctgcctgtgatggtgacccttgtgccttattggatggtatatttccagatgcagtccacctatgtcctccaaggtctccacctccatatccccaacatcttcaggaccaaccctaacatctctttgttgctgagatcagatagcagtaactacaggatcccagaagcctggctcctactggccaatgttgcggtgatcctgattctgatccctgtcaaggatcacttgattgatcctctgctgctgcggtgcaagctgctgccctcatctctgcagaaaatggccctgggcatgttctttgggttcacctccatcattgtggcaggagtcctggagaaagaacgcttacagtacattgctgccaaccagacagtgcctcagctgattgggaaggacttatactatgcagcgccactgtctatctggtggcagatcccccagtacctgctcatcggggtcagtgagatctttgccagcatcccaggtctggagttcgcttactcagaagccccacgctccatgcagggtgcaattatgggcatcttcttctgcctttccggggtgggctcactgctaggctctggcttggtggccctactgtcttttcctgggggctggatgtactgccccaaggactttgggaacatcaacaactgtcagatggaccgttacttcttcctgctggccggcattgaggctgtcacagccgtcctgtttctctggattgctggtcgctatgagaggactcgccaggacccagcctcccagcgctcgtccagcagggtcagaggcaccggtggatcctacccatacgatgttccagattacgctctcgagagcaccggtggcagcggagctactaacttcagcctgctgaagcaggctggagacgtggaggagaaccctggacctgccggtatggtgagcaagggcgaggaggataacatggccatcatcaaggagttcatgcgcttcaaggtgcacatggagggctccgtgaacggccacgagttcgagatcgagggcgagggcgagggccgcccctacgagggcacccagaccgccaagctgaaggtgaccaagggtggccccctgcccttcgcctgggacatcctgtcccctcagttcatgtacggctccaaggcctacgtgaagcaccccgccgacatccccgactacttgaagctgtccttccccgagggcttcaagtgggagcgcgtgatgaacttcgaggacggcggcgtggtgaccgtgacccaggactcctccctgcaggacggcgagttcatctacaaggtgaagctgcgcggcaccaacttcccctccgacggccccgtaatgcagaagaagaccatgggctgggaggcctcctccgagcggatgtaccccgaggacggcgccctgaagggcgagatcaagcagaggctgaagctgaaggacggcggccactacgacgctgaggtcaagaccacctacaaggccaagaagcccgtgcagctgcccggcgcctacaacgtcaacatcaagttggacatcacctcccacaacgaggactacaccatcgtggaacagtacgaacgcgccgagggccgccactccaccggcggcatggacgagctgtacaagtaa

1. **pCAG-Slc15a4-FLAG-2A-mCherry-pA**

atggagggcgagcgggcgccgctgttgggttcgcggcgtccggcagtgtcggcggcgtccgcggtgttcgcggggcggcgtgcggcgtgcggggcggtgctgttggccgagctgctggagcgcgcggccttctacggcgtcacggccaacctggtgctgttcctgaatggcgcgccgttcgactgggagggcgcgcaggccagccaggcgctgctgctcttcatgggcctcacctatctgggctcgccgttcgggggctggctcgccgacgcccgcctcggccgggcccgcgccatcctgctcagcctggccctctacctgctgggcctgctggccttcccgctgctggccgcgccccgctcgcgctccttcctctgcggggacccgcggcccgagctcgtgcgcaactgctcggcgcccttccccaacgggtcagcgtcgtgtcccgagaacgccgcgcgccgctgtgcccccgccaccttcgcggggctcgtgctcgtgggtctcggggtggccacggtcaaggccaacatcacgcccttcggcgccgatcaggttaaagatcgaggtccagaagccactcggagatttttcaattggttttactggagcattaatttgggagcaatcctgtcattaggaggtattgcctatattcagcagaatgtgagctttttcacaggctacctgattcccacagtctgtgtggccattgctttcctggtcttcctctgtggccagagtgtcttcatcaccaagcctcctgacggcagtgccttcactgacatgttcagaattctgacctacagttgctgctcccagagaggagggcagcggagaagtggtgaaggccttggagtctttcagcaatcttctaaacacagtctgtttgattcatgtaagatgtcgcgtggagggccgttcacagaggacaaagtggaagacgtgaaagccctggtcaagattgtgcccgtgttcttggctctgattccttactggacagtgtatttccaaatgcagaccacatatgctttacagagtcttcatttgaagattccagaaatctcaagtatcaccaccacccatcacacgctgcctgcagcttggctcaccatgtttgatgcggtgctcatcctcctgctcatcccactgaaggacaagctggtggacccggtgctgaggagacatggcctgctcccatcctccctgaagaggattgctgtggggatgttcttcgtcatgtgctctgcctttgctgcaggaatcctggagagtaaaaggctggacctggtaaaggagaagaccattaaccagaccattggtggtgtggtgtaccatgctgctgacttgcccatctggtggcagatcccacagtatgtgctcattggcatcagtgagatatttgcaagtatagcaggtctggaatttgcatactcagctgcccccaagtccatgcagagcgccatcatgggactcttcttcttcttctctggcattgggtcctttgtgggctcaggactgttggccctggtgtctctcaaggccattgggtggatgagcagtcatacagattttgggaacatcaacagctgccacctgcattactacttcttcctgctggccgccatccagggagctacgctgctgctcttcctcattgtgtctgtgaagtacgaccgccagcgagccaggacagatggggggccagccagcacgaggaccaccggtggatccgactacaaagacgatgacgacaagctcgagagcaccggtggcagcggagctactaacttcagcctgctgaagcaggctggagacgtggaggagaaccctggacctgccggtatggtgagcaagggcgaggaggataacatggccatcatcaaggagttcatgcgcttcaaggtgcacatggagggctccgtgaacggccacgagttcgagatcgagggcgagggcgagggccgcccctacgagggcacccagaccgccaagctgaaggtgaccaagggtggccccctgcccttcgcctgggacatcctgtcccctcagttcatgtacggctccaaggcctacgtgaagcaccccgccgacatccccgactacttgaagctgtccttccccgagggcttcaagtgggagcgcgtgatgaacttcgaggacggcggcgtggtgaccgtgacccaggactcctccctgcaggacggcgagttcatctacaaggtgaagctgcgcggcaccaacttcccctccgacggccccgtaatgcagaagaagaccatgggctgggaggcctcctccgagcggatgtaccccgaggacggcgccctgaagggcgagatcaagcagaggctgaagctgaaggacggcggccactacgacgctgaggtcaagaccacctacaaggccaagaagcccgtgcagctgcccggcgcctacaacgtcaacatcaagttggacatcacctcccacaacgaggactacaccatcgtggaacagtacgaacgcgccgagggccgccactccaccggcggcatggacgagctgtacaagtaa

1. **pCAG-TASL-AausFP1-pA**

atgggcctatcagaaggatatctcagtggacttacctactggaatgacattcattggaattgtgcatcttataatgaaccggtggctggggaccaaggcaaagagacaagttctgttgctgctctttcatattcctctgtggatgaaacacaagttcaaagtctttatgtgagctgcaaatcctctgggaagtttatttcatcagtgcatgcaagggcgagtcagcacagcagaagccagagcagaacagtgctgcaggcaaacagcaaccctgtatttgaaagtccaactttagctgcagttggtatatgcagagatgtgatcagggagacctacttggttccaccttcttgtaaaagtatttgcaaaaattacaacgacttacatattgcagggggacaggtgatggccattaactcagtaatggcaaatttcccctctgagagcagctttgaagatggtcctttgctaaagtcatctgagatttctttgtccatggaggattccacttccactcagctcactgaacttcccctcaaacctatccagcggtactcatcctactggaggataaccagcatcaaagagaaaagcagcctgcaaatgcagaagcctatttcaaatgcagtgctcaatgagtacctggagcagaaggtggtggaattgtataagcaatatattatggacactgtgtttcatgacagttctcctacccagattctggcatcagaattcatcatgacgaatgtagatcaaattagtcttcaagtgtctaaagagaagaacctggacacttcaaaagtcaaggacatagttattagccacctgttgcagttggtatcatctgagatcagcacccctagtcttcatatttctcagtatagcaatataactccaggcggaggagggagcatgagctatggtgccctgctgtttcgtgaaaaaattccatacgtggttgaaatggagggtgacgttgaaggtatgaaattcagcgttcgtggtaaaggtcatggtgacgctaacactggtaaaatcgaggcgtctttcatttgcaccactggtgaactgccagttccatggtctagcatcctgaccactgttacctacggtgcacagtgtttcgcgaaatatccgaacgacattaaggactatccaaagtctgcgatgccggaaggttacgtccaggaacgtactatcaccttcgaaaacgatggtgtatataagactcgtgcggaagttacctacgagaaaggttccgtttacaaccgtgttaccctgaacggcagcggctttaaaaaaggcggcaatatcctgggtaaaaagctggaatttaattataacccgcactgcatctatgttctgccagacgttcaaaacaacggcattaaatgctacattaacatcgttcacgacgtaatcggtggcggtcagattattgcggctcatcagcagctgaacaccccactgggcggtggtcctgtagacatcccgcactatcaccacatccaggctcacaccattctgtccaaagacccgaaagaaactcgcgaccacatgaacgtcgtcgaagttttccgtgcgatcgactgtaaaaccgcctacgcttaa

1. **pCAG-Slc15a3-BFP-pA**

atggggtctgcccctcgcgcggaagagcaaccaagcaggtccggagagcgacaaccgctggtggcccgaggcccgcggggaccacgacggtggagacggacagctgctgctgcggtgctgctagtgcagatgctggaacgcgctgccttctttggtgtcacctccaacctcgtgctctacctcaacagcttgaacttcaattgggacggccaacaggcatcacgggccacactcctcttcctaggcgcctcctacctgctggctccagtggggggctggctggcagacgtgtacctgggacgcttcctcactatctcgctcagcctgctgctctatctggctgcctctggcttgttgctcaccaccatcaccaatgatggccgcagatccttctgtggagagatgcccgagttgccactggaacctgcctgcccatcctcaagctgccaaggctcctggtccagcccctactgcgcgaccaccctctacttggtgttgctgctcctggccctggctgccagctccgtcaggagcaccctcacctcgttcggggctgaccaggtgatggatctcgggcgcgatgccacccgccgcttcttcaactggttctattggagcatcaacctgggtgccatattgtccctgttggtggtggctttcatcgagcagaacatcagcttcctatggggctacagcatcatcgtgggcctcgtgggcctggcattcttcatttttctctttgccacaccagtcttcatcaccaagcccccaacaggcagccaagtgtcatctatgctgaagcttgcgttccaaaactgctgtccctgccggaggtcctcttccagggactctgaaagtgcccacctgttgcctgaccagaggtctaaccagcctggaccttctccacaagaagatatggccaacttccaggtgctggtgaagatcctgcctgtgatggtgacccttgtgccttattggatggtatatttccagatgcagtccacctatgtcctccaaggtctccacctccatatccccaacatcttcaggaccaaccctaacatctctttgttgctgagatcagatagcagtaactacaggatcccagaagcctggctcctactggccaatgttgcggtgatcctgattctgatccctgtcaaggatcacttgattgatcctctgctgctgcggtgcaagctgctgccctcatctctgcagaaaatggccctgggcatgttctttgggttcacctccatcattgtggcaggagtcctggagaaagaacgcttacagtacattgctgccaaccagacagtgcctcagctgattgggaaggacttatactatgcagcgccactgtctatctggtggcagatcccccagtacctgctcatcggggtcagtgagatctttgccagcatcccaggtctggagttcgcttactcagaagccccacgctccatgcagggtgcaattatgggcatcttcttctgcctttccggggtgggctcactgctaggctctggcttggtggccctactgtcttttcctgggggctggatgtactgccccaaggactttgggaacatcaacaactgtcagatggaccgttacttcttcctgctggccggcattgaggctgtcacagccgtcctgtttctctggattgctggtcgctatgagaggactcgccaggacccagcctcccagcgctcgtccagcagggtcagaggcggcggaggagggagcatgagcgagctgattaaggagaacatgcacatgaagctgtacatggagggcaccgtggacaaccatcacttcaagtgcacatccgagggcgaaggcaagccctacgagggcacccagaccatgagaatcaaggtggtcgagggcggccctctccccttcgccttcgacatcctggctactagcttcctctacggcagcaagaccttcatcaaccacacccagggcatccccgacttcttcaagcagtccttccctgagggcttcacatgggagagagtcaccacatacgaggacgggggcgtgctgaccgctacccaggacaccagcctccaggacggctgcctcatctacaacgtcaagatcagaggggtgaacttcacatccaacggccctgtgatgcagaagaaaacactcggctgggaggccttcaccgagacgctgtaccccgctgacggcggcctggaaggcagaaacgacatggccctgaagctcgtgggcgggagccatctgatcgcaaacatcaagaccacatatagatccaagaaacccgctaagaacctcaagatgcctggcgtctactatgtggactacagactggaaagaatcaaggaggccaacaacgagacctacgtcgagcagcacgaggtggcagtggccagatactgcgacctccctagcaaactggggcacaagcttaattaa

1. **pCAG-Slc15a4-smURFP-pA**

atggagggcgagcgggcgccgctgttgggttcgcggcgtccggcagtgtcggcggcgtccgcggtgttcgcggggcggcgtgcggcgtgcggggcggtgctgttggccgagctgctggagcgcgcggccttctacggcgtcacggccaacctggtgctgttcctgaatggcgcgccgttcgactgggagggcgcgcaggccagccaggcgctgctgctcttcatgggcctcacctatctgggctcgccgttcgggggctggctcgccgacgcccgcctcggccgggcccgcgccatcctgctcagcctggccctctacctgctgggcctgctggccttcccgctgctggccgcgccccgctcgcgctccttcctctgcggggacccgcggcccgagctcgtgcgcaactgctcggcgcccttccccaacgggtcagcgtcgtgtcccgagaacgccgcgcgccgctgtgcccccgccaccttcgcggggctcgtgctcgtgggtctcggggtggccacggtcaaggccaacatcacgcccttcggcgccgatcaggttaaagatcgaggtccagaagccactcggagatttttcaattggttttactggagcattaatttgggagcaatcctgtcattaggaggtattgcctatattcagcagaatgtgagctttttcacaggctacctgattcccacagtctgtgtggccattgctttcctggtcttcctctgtggccagagtgtcttcatcaccaagcctcctgacggcagtgccttcactgacatgttcagaattctgacctacagttgctgctcccagagaggagggcagcggagaagtggtgaaggccttggagtctttcagcaatcttctaaacacagtctgtttgattcatgtaagatgtcgcgtggagggccgttcacagaggacaaagtggaagacgtgaaagccctggtcaagattgtgcccgtgttcttggctctgattccttactggacagtgtatttccaaatgcagaccacatatgctttacagagtcttcatttgaagattccagaaatctcaagtatcaccaccacccatcacacgctgcctgcagcttggctcaccatgtttgatgcggtgctcatcctcctgctcatcccactgaaggacaagctggtggacccggtgctgaggagacatggcctgctcccatcctccctgaagaggattgctgtggggatgttcttcgtcatgtgctctgcctttgctgcaggaatcctggagagtaaaaggctggacctggtaaaggagaagaccattaaccagaccattggtggtgtggtgtaccatgctgctgacttgcccatctggtggcagatcccacagtatgtgctcattggcatcagtgagatatttgcaagtatagcaggtctggaatttgcatactcagctgcccccaagtccatgcagagcgccatcatgggactcttcttcttcttctctggcattgggtcctttgtgggctcaggactgttggccctggtgtctctcaaggccattgggtggatgagcagtcatacagattttgggaacatcaacagctgccacctgcattactacttcttcctgctggccgccatccagggagctacgctgctgctcttcctcattgtgtctgtgaagtacgaccgccagcgagccaggacagatggggggccagccagcacgaggaccggcggaggagggagctctagaatggctaaaacttccgaacaacgcgtgaacattgcgaccctgctgaccgaaaacaaaaaaaaaattgtagacaaagcgagccaagatctgtggcgtcgtcatccagatctgattgccccgggtggtattgcatttagccagcgtgaccgtgcactgtgcctgcgtgattatggttggtttctgcacctgatcacgttctgcctgctggcaggtgataaaggtccgattgaatctatcggcctgattagcattcgcgaaatgtacaactctctgggtgttccggttccagcaatgatggaatctatccgctgtctgaaagaagcaagcctgtccctgctggatgaagaggacgcaaatgaaacggccccatacttcgactacatcatcaaagctatgagctaa

1. **Alkbh5^H205A^**

atggcggccgccagcggctacaccgacctgcgggagaagctcaagtccatgacgtcccgggacaactacaaggcgggcagtcgggaggccgccgccgctgcggccgccgccgtggctgccgctgccgctgccgcggccgccgctgagccttacccggcgtccgggaccaccaagcggaaataccaggaggactcggaccccgagcgcagcgactacgaggagcatcagttgcagaaggaggaagaggcgcgcaaggtgaagagcggcatccggcagatccggctcttcagtcaggatgagtgctccaagatcgaggcccgcatcgatgaggtggtgtcccgcgccgagaagggcctgtacaacgagcacacggtggaccgggcccccctgcgcaacaagtacttcttcggcgagggctacacgtacggggcccagctgcagaagcgcgggccgggccaggagcgcctctacccgccgggcgacgtcgacgagatcccggactgggtgcatcagctggtgatccagaagctggtggagcaccgcgtcatccccgagggcttcgtcaacagcgcggtcatcaacgactaccagcccggcggctgcatcgtgtccca/gccgttgaccccatccacatcttcgagcgccccatcgtgtccgtgtctttcttcagcgactcggcactttgcttcggctgcaagttccagttcaagcccatccgggtgtcggaacctgtgctttctctgccggtgcgcagggggagcgtgactgtgctcagtgggtatgctgctgatgaaatcactcactgcatacggcctcaggacattaaggaacgccgggcggtcatcattctcaggaagacaagattagatgcaccgcgattggaaacaaaatccctgagcagctccacattgccacccagctatgcttcagatcgcctgtcaggaaacaccagagaccctgcgctgaaacccaaaaggtcccaccgcaaggcagaccctgatgctgcccacaggccccggatcctggaaatggacaaagaagaaaaccggcggtctgtgctcctgcccacacaccggcggagggggagttttagctctgagaactattggcgcaaatcctatgagtcctcggaagattgcccagaggcagccagcagccccacccgcaaggtgaagatgaggagacac

1. **Mettl3^D395A&W398A^**

atggggtcggacacgtggagctctatccaggcccataagaaacagctggactcgcttcgcgagagattgcagcggcgacggaagcaggactctgggcacttggatttaaggaacccagaagcggcactgtccccaaccttccgtagtgatagtcccgtgcctactgcccctacctctagcggccctaagcccagcacaacatctgtggcccctgaactagctacagaccctgagttagagaagaagttgctacaccacctctcagatctggccttgaccttgcccactgatgctgtttccatccgtcttgccatctctacgccagatgcacctgccactcaagatggggtagaaagccttctccagaaatttgccgcccaggagttgattgaggtaaagcgaggtctcctccaagatgatgcacatcctactcttgtaacttacgctgaccactccaagctgtctgccatgatgggggctgtggcagaaaagaaaggtcttggagaggtagcagggaccatcgcagggcagaaacggcgtgcagaacaggatttgactacagtgaccacctttgccagctctttagcatctggtctggcctcttcagcatcagaaccagctaaggagccggctaagaagtcaaggaagcacgctgcctccgatgttgatctggagatagaaagccttttgaaccaacagtcaacgaaagaacagcagagcaagaaggtcagtcaggagatcctagagctattaaataccacaacagccaaggaacagtccattgttgaaaagtttcgctctcgaggtcgggcccaggtgcaagaattttgtgattatgggaccaaggaagagtgcatgaaagccagtgacgctgaccggccttgtcgcaagctgcacttcagacgaattatcaataagcacactgatgaatctttaggtgactgctctttccttaacacatgtttccacatggacacctgcaaatatgttcactatgaaattgatgcttgtgttgattctgagagtcctggcagcaaggagcatatgccaagccaggagcttgctcttacacagagtgttgggggtgactccagtgctgatcgactctttccacctcagtggatctgttgtgatatccgctacctggacgtcagtatcttgggcaaatttgcagttgtgatggctga/ccccaccttg/gcggatattcacatggagctaccgtatgggacattaacagatgatgagatgcgcaggctcaatataccagtgctacaggatgacggctttcttttcctctgggtcacaggaagggccatggaattgggcagagaatgtctgaacctctggggttatgaacgggtggatgaaatcatctgggtgaagactaatcagctgcagcgcatcattaggacgggccggacgggtcactggttaaaccacgggaaggaacactgcttggttggtgttaagggaaatcctcaaggattcaaccagggcctggactgcgatgtgattgtagctgaggttcgttccaccagtcataaaccagatgaaatatatggcatgattgagagactgtcccctggcacccgcaagattgagttatttggacgaccacacaatgtgcagcccaactggattactcttggaaaccaactggatgggatacacctactagacccagatgtggttgccaggtttaagcaaaggtatccggacggcatcatctctaaacctaagaattta

"WT (Green)"/"Mutant (Red)"
